# Supplementary material for: Genome-wide identification of SHMT family genes in C3, C3-C4, and C4 Salsoleae s.l. species
Source: PeerJ. 2025 Sep 3;13:e19978. doi: 10.7717/peerj.19978 (PMC12422262; doi:10.7717/peerj.19978)
Supplement: Supplemental Information 1 [file peerj-13-19978-s001.zip › Supplemental Raw Data/Explanation of the MIQE Checklist.docx]

**EXPERIMENTAL DESIGN**

**Number within each group:** The control group is defined as the average expression level of the roots of Salsola junatovii, while the treatment group consists of the expression levels of the roots, stems, and leaves from four species of the Salsoleae tribe, with 3 replicates per tissue.

**NUCLEIC ACID EXTRACTION**

**Yield:** Yield ranged from 271.1 ng/µL to 1207.8 ng/µL

**Inhibition testing (Cq dilutions, spike or other):** Based on the previous experience of the research group, it was found that by performing 3-, 4-, and 5-fold dilutions of the cDNA samples, the best results were achieved with a 4-fold dilution, and no PCR inhibition was detected.

**REVERSE TRANSCRIPTION**

**Amount of RNA and reaction volume:** The amount of RNA used in each reaction (after reverse transcription to cDNA) should be less than 100 ng, and the reaction volume is 25 µL.

**Priming oligonucleotide (if using GSP) and concentration:** The forward and reverse primers for PCR are 10 µM in each reaction.

**Reverse transcriptase and concentration:** The reverse transcription was performed using PrimeScript^TM^ RT Reagent Kit with gDNA Eraser, with 1.0 μL of PrimeScript RT Enzyme Mix Ⅰ added per 20 μL reaction volume.

**Temperature and time:** The reverse transcription reaction was carried out at 37°C for 15 minutes, followed by 5 seconds at 85°C and then stored at 4°C.

**Manufacturer of reagents and catalogue numbers:** The reagent kit used is PrimeScript^TM^ RT Reagent Kit with gDNA Eraser, manufactured by Takara Bio, with catalogue number Code No. RR047Q.

**Storage conditions of cDNA:** All cDNA samples were stored at 4°C.

**qPCR TARGET INFORMATION**

**Sequence accession number:** The sequence accession numbers for the genes of all species used in the experiments have not been published. However, the protein sequence accession numbers can be found in the Supplemental Raw Data file, SHMT_protein_sequences.docx.

**Location of amplicon:** The amplicon positions for the SHMT genes are as follows: SjSHMT1 has an amplicon from 665 to 755, SjSHMT2 from 885 to 1001, SjSHMT4 from 91 to 220, and SjSHMT7 from 52 to 175. For OlSHMT, the amplicons are as follows: OlSHMT1 from 48 to 148, OlSHMT2 from 238 to 360, OlSHMT3 from 1247 to 1346, OlSHMT4 from 97 to 222, and OlSHMT7 from 259 to 326. For SfSHMT, the amplicons are: SfSHMT1 from 66 to 131, SfSHMT2 from 522 to 651, SfSHMT4 from 3 to 97, and SfSHMT7 from 261 to 342. Lastly, for XaSHMT, the amplicons are: XaSHMT1 from 94 to 202, XaSHMT2 from 273 to 384, XaSHMT3 from 63 to 136, XaSHMT4 from 825 to 938, and XaSHMT7 from 279 to 334.

**Amplicon lengthThe amplicon lengths for the SHMT genes are as follows:** SjSHMT1 has an amplicon length of 90, SjSHMT2 has 116, SjSHMT4 has 129, and SjSHMT7 has 123. For OlSHMT, the amplicon lengths are as follows: OlSHMT1 has 100, OlSHMT2 has 122, OlSHMT3 has 99, OlSHMT4 has 125, and OlSHMT7 has 67. For SfSHMT, the amplicon lengths are: SfSHMT1 has 65, SfSHMT2 has 129, SfSHMT4 has 94, and SfSHMT7 has 81. Lastly, for XaSHMT, the amplicon lengths are: XaSHMT1 has 108, XaSHMT2 has 111, XaSHMT3 has 73, XaSHMT4 has 113, and XaSHMT7 has 55.

**In silico specificity screen (BLAST, etc):** Primer design and in silico specificity screening were performed using NCBI tools, including BLAST. The primers were found to have high specificity to the target gene sequence, with no significant off-target matches identified in the database.

**Sequence alignment: The genome data used is unpublished:** however, primer design was performed by aligning with a closely related species, Chenopodium (quinoa), within the same family.

**Secondary structure analysis of amplicon:** The analysis was performed using NCBI and DNAMAN, and no amplicon showed any hairpin structures that would lead to amplification failure.

**qPCR PROTOCOL**

**Complete reaction conditions/Reaction volume and amount of cDNA/DNA/Primer, (probe), Mg++ and dNTP concentrations/Polymerase identity and concentration:** The reaction system consists of a total volume of 25 µL, containing 1 µL of forward and reverse primers, 2 µL of template, 8.5 µL of sterilized water, and 12.5 µL of enzyme. The enzyme used is TB Green Premix Ex Taq Ⅱ. The qPCR cycling conditions are as follows: pre-incubation at 95°C for 30 seconds, followed by the PCR amplification stage at 95°C for 5 seconds and 60°C for 30 seconds. This process is repeated for 40 cycles. In the melt curve stage, the conditions are 95°C for 10 seconds, 60°C for 30 seconds, and 95°C for 1 second.

**Buffer/kit identity and manufacturer:** The reagent kit used is the TB Green Premix Ex Taq ^TM^ Ⅱ kit from TaKaRa, with catalogue number Code No. RR820A.

**Additives (SYBR Green I, DMSO, etc.):** TB Green Premix Ex Taq Ⅱ is an enzyme with fluorescence.

**Manufacturer of plates/tubes and catalog number:** The PCR 8-strip tubes used are from LABSELECT, model PST-0208-FT-C, with caps.

**Complete thermocycling parameters:** The enzyme used is TB Green Premix Ex Taq Ⅱ. The qPCR cycling conditions are as follows: pre-incubation at 95°C for 30 seconds, followed by the PCR amplification stage at 95°C for 5 seconds and 60°C for 30 seconds. This process is repeated for 40 cycles. In the melt curve stage, the conditions are 95°C for 10 seconds, 60°C for 30 seconds, and 95°C for 1 second.

**Reaction setup (manual/robotic):** Manual pipetting of all reagents

**Manufacturer of qPCR instrument:** QuantStudio Design & Analysis SC Software v1.0.0

**DATA ANALYSIS.**

**qPCR analysis program (source, version):** Using QuantStudio Design & Analysis SC Software v1.0.0, provided by Applied Biosystems (by Thermo Fisher Scientific).

**Cq method determination:** Threshold Method

**Outlier identification and disposition:** Each gene was tested in 6 experiments, and the 3 normal data points with the smallest standard deviation were selected for analysis.

**Justification of number and choice of reference genes:** β-actin was chosen as the reference gene due to its stable expression in the roots, stems, and leaves of various species.

**Software (source, version):** Manufacturer of qPCR instrument: QuantStudio Design & Analysis SC Software v1.0.0
