# Supplementary material for: Genome-wide identification of SHMT family genes in C3, C3-C4, and C4 Salsoleae s.l. species
Source: PeerJ. 2025 Sep 3;13:e19978. doi: 10.7717/peerj.19978 (PMC12422262; doi:10.7717/peerj.19978)
Supplement: Supplemental Information 1 [file peerj-13-19978-s001.zip › Supplemental Raw Data/raw data Table.docx]

Raw data-Table1. CT values of three tissues from four species of Salsoleae

|  | Root | | | | Stem | | | | Leaf | | | |
| --- | --- | --- | --- | --- | --- | --- | --- | --- | --- | --- | --- | --- |
| Gene name | CT | SE | actin-CT | SE | CT | SE | actin-CT | SE | CT | SE | actin-CT | SE |
| *SfSHMT1* | 25.991 | 0.175 | 19.500 | 0.045 | 21.532 | 0.009 | 18.871 | 0.030 | 21.994 | 0.030 | 20.945 | 0.015 |
|  | 25.411 |  | 19.542 |  | 21.517 |  | 18.900 |  | 21.981 |  | 20.915 |  |
|  | 25.852 |  | 19.390 |  | 21.547 |  | 18.799 |  | 21.898 |  | 20.965 |  |
| *SfSHMT2* | 22.142 | 0.080 | 19.500 | 0.045 | 21.906 | 0.015 | 18.871 | 0.030 | 25.789 | 0.124 | 20.945 | 0.015 |
|  | 22.276 |  | 19.542 |  | 21.956 |  | 18.900 |  | 25.484 |  | 20.915 |  |
|  | 22.418 |  | 19.390 |  | 21.943 |  | 18.799 |  | 25.899 |  | 20.965 |  |
| *SfSHMT4* | 22.504 | 0.012 | 19.500 | 0.045 | 22.490 | 0.043 | 18.871 | 0.030 | 23.690 | 0.075 | 20.945 | 0.015 |
|  | 22.532 |  | 19.542 |  | 22.349 |  | 18.900 |  | 23.725 |  | 20.915 |  |
|  | 22.543 |  | 19.390 |  | 22.374 |  | 18.799 |  | 23.930 |  | 20.965 |  |
| *SfSHMT7* | 22.111 | 0.026 | 19.500 | 0.045 | 21.121 | 0.096 | 18.871 | 0.030 | 22.719 | 0.049 | 20.945 | 0.015 |
|  | 22.034 |  | 19.542 |  | 21.288 |  | 18.900 |  | 22.719 |  | 20.915 |  |
|  | 22.113 |  | 19.390 |  | 21.452 |  | 18.799 |  | 22.866 |  | 20.965 |  |
| *SjSHMT1* | 20.815 | 0.071 | 17.471 | 0.062 | 20.759 | 0.010 | 16.641 | 0.059 | 22.524 | 0.053 | 19.010 | 0.229 |
|  | 20.917 |  | 17.511 |  | 20.792 |  | 16.452 |  | 22.365 |  | 19.029 |  |
|  | 21.061 |  | 17.675 |  | 20.767 |  | 16.477 |  | 22.523 |  | 19.706 |  |
| *SjSHMT2* | 29.557 | 0.160 | 17.471 | 0.062 | 26.787 | 0.019 | 16.641 | 0.059 | 25.609 | 0.081 | 19.010 | 0.229 |
|  | 30.109 |  | 17.511 |  | 26.806 |  | 16.452 |  | 25.845 |  | 19.029 |  |
|  | 29.798 |  | 17.675 |  | 26.850 |  | 16.477 |  | 25.857 |  | 19.706 |  |
| *SjSHMT4* | 23.189 | 0.035 | 17.471 | 0.062 | 22.779 | 0.067 | 16.641 | 0.059 | 23.832 | 0.086 | 19.010 | 0.229 |
|  | 23.147 |  | 17.511 |  | 22.546 |  | 16.452 |  | 23.996 |  | 19.029 |  |
|  | 23.268 |  | 17.675 |  | 22.676 |  | 16.477 |  | 23.700 |  | 19.706 |  |
| *SjSHMT7* | 22.111 | 0.020 | 17.471 | 0.062 | 22.025 | 0.120 | 16.641 | 0.059 | 22.668 | 0.071 | 19.010 | 0.229 |
|  | 22.076 |  | 17.511 |  | 22.243 |  | 16.452 |  | 22.455 |  | 19.029 |  |
|  | 22.040 |  | 17.675 |  | 22.441 |  | 16.477 |  | 22.454 |  | 19.706 |  |
| *XaSHMT1* | 22.614 | 0.106 | 18.912 | 0.056 | 18.990 | 0.047 | 17.279 | 0.091 | 19.804 | 0.020 | 18.611 | 0.151 |
|  | 22.953 |  | 18.728 |  | 18.966 |  | 17.564 |  | 19.736 |  | 18.360 |  |
|  | 22.661 |  | 18.765 |  | 18.838 |  | 17.541 |  | 19.760 |  | 18.884 |  |
| *XaSHMT2* | 25.726 | 0.012 | 18.912 | 0.056 | 22.358 | 0.051 | 17.279 | 0.091 | 23.982 | 0.072 | 18.611 | 0.151 |
|  | 25.733 |  | 18.728 |  | 22.492 |  | 17.564 |  | 24.056 |  | 18.360 |  |
|  | 25.766 |  | 18.765 |  | 22.526 |  | 17.541 |  | 23.814 |  | 18.884 |  |
| *XaSHMT3* | 27.162 | 0.064 | 18.912 | 0.056 | 24.889 | 0.053 | 17.279 | 0.091 | 24.995 | 0.029 | 18.611 | 0.151 |
|  | 27.213 |  | 18.728 |  | 25.046 |  | 17.564 |  | 24.905 |  | 18.360 |  |
|  | 27.373 |  | 18.765 |  | 24.887 |  | 17.541 |  | 24.988 |  | 18.884 |  |
| *XaSHMT4* | 26.096 | 0.060 | 18.912 | 0.056 | 20.965 | 0.015 | 17.279 | 0.091 | 20.283 | 0.055 | 18.611 | 0.151 |
|  | 26.096 |  | 18.728 |  | 20.948 |  | 17.564 |  | 20.251 |  | 18.360 |  |
|  | 26.275 |  | 18.765 |  | 20.913 |  | 17.541 |  | 20.103 |  | 18.884 |  |
| *XaSHMT7* | 26.148 | 0.041 | 18.912 | 0.056 | 22.245 | 0.157 | 17.279 | 0.091 | 22.794 | 0.030 | 18.611 | 0.151 |
|  | 26.204 |  | 18.728 |  | 22.528 |  | 17.564 |  | 22.769 |  | 18.360 |  |
|  | 26.289 |  | 18.765 |  | 22.789 |  | 17.541 |  | 22.694 |  | 18.884 |  |
| *OlSHMT1* | 22.453 | 0.030 | 18.120 | 0.183 | 20.880 | 0.066 | 19.719 | 0.119 | 17.694 | 0.126 | 20.328 | 0.189 |
|  | 22.355 |  | 18.674 |  | 20.934 |  | 19.407 |  | 17.275 |  | 19.760 |  |
|  | 22.438 |  | 18.132 |  | 21.099 |  | 19.328 |  | 17.374 |  | 19.765 |  |
| *OlSHMT2* | 21.151 | 0.035 | 18.120 | 0.183 | 22.148 | 0.045 | 19.719 | 0.119 | 23.603 | 0.074 | 20.328 | 0.189 |
|  | 21.256 |  | 18.674 |  | 22.016 |  | 19.407 |  | 23.434 |  | 19.760 |  |
|  | 21.258 |  | 18.132 |  | 22.009 |  | 19.328 |  | 23.687 |  | 19.765 |  |
| *OlSHMT3* | 24.254 | 0.006 | 18.120 | 0.183 | 26.112 | 0.048 | 19.719 | 0.119 | 25.649 | 0.084 | 20.328 | 0.189 |
|  | 24.240 |  | 18.674 |  | 25.949 |  | 19.407 |  | 25.928 |  | 19.760 |  |
|  | 24.260 |  | 18.132 |  | 26.062 |  | 19.328 |  | 25.865 |  | 19.765 |  |
| *OlSHMT4* | 22.854 | 0.302 | 18.120 | 0.183 | 22.947 | 0.121 | 19.719 | 0.119 | 21.296 | 0.236 | 20.328 | 0.189 |
|  | 23.697 |  | 18.674 |  | 22.605 |  | 19.407 |  | 21.977 |  | 19.760 |  |
|  | 23.810 |  | 18.132 |  | 22.986 |  | 19.328 |  | 22.028 |  | 19.765 |  |
| *OlSHMT7* | 22.099 | 0.327 | 18.120 | 0.183 | 23.855 | 0.005 | 19.719 | 0.119 | 23.200 | 0.026 | 20.328 | 0.189 |
|  | 23.026 |  | 18.674 |  | 23.843 |  | 19.407 |  | 23.283 |  | 19.760 |  |
|  | 23.126 |  | 18.132 |  | 23.860 |  | 19.328 |  | 23.271 |  | 19.765 |  |
| *Sj*: *Salsola junatovii*, *Ol*: *Oreosalsola laricifolia*, S*f*: *Soda foliosa*, *Xa*: *Xylosalsola arbuscula*, *SHMT*: Serine Hydroxymethyltransferase. | | | | | | | | | | | |  |

Raw data-Table2. Relative expression values for each tissue in each species

| Gene name | Root | Stem | Leaf |
| --- | --- | --- | --- |
| *SjSHMT1* | 8.44 | 4.61 | 9.40 |
| *SjSHMT2* | 0.02 | 0.07 | 0.95 |
| *SjSHMT4* | 1.75 | 1.24 | 3.63 |
| *SjSHMT7* | 3.81 | 1.67 | 9.05 |
| *OlSHMT1* | 1.32 | 8.13 | 129.15 |
| *OlSHMT2* | 3.02 | 3.83 | 1.85 |
| *OlSHMT3* | 0.37 | 0.24 | 0.39 |
| *OlSHMT4* | 0.64 | 2.22 | 6.47 |
| *OlSHMT7* | 1.05 | 1.10 | 2.31 |
| *SfSHMT1* | 0.17 | 2.01 | 6.35 |
| *SfSHMT2* | 1.84 | 1.52 | 0.47 |
| *SfSHMT4* | 1.55 | 1.10 | 1.79 |
| *SfSHMT7* | 2.11 | 2.38 | 3.62 |
| *XaSHMT1* | 7.35 | 40.73 | 50.92 |
| *XaSHMT2* | 0.92 | 3.53 | 2.80 |
| *XaSHMT3* | 0.32 | 0.63 | 1.39 |
| *XaSHMT4* | 0.69 | 10.11 | 37.39 |
| *XaSHMT7* | 0.66 | 3.38 | 6.43 |
| *Sj*: *Salsola junatovii*, *Ol*: *Oreosalsola laricifolia*, S*f*: *Soda foliosa* , *Xa*: *Xylosalsola arbuscula*, *SHMT*: Serine Hydroxymethyltransferase, Using the average CT value of the roots for each species as the control group, relative expression levels were calculated using the 2^–ΔΔCT^ method. | | | |

Raw data-Table3.Transcriptional expression levels of *SHMT* in 31 species

| Genus/Tribe | *Gene* | TPM |
| --- | --- | --- |
| *Alternanthera* | *AbraSHMT1* | 1159.37 |
|  | *AbraSHMT2* | 7.94514 |
|  | *AbraSHMT3* | 14.5235 |
|  | *AbraSHMT4* | 334.96 |
|  | *AbraSHMT7* | 27.7758 |
|  | *AsesSHMT1* | 1007.41 |
|  | *AsesSHMT3* | 5.94804 |
|  | *AsesSHMT4* | 210.001 |
|  | *AsesSHMT7* | 18.2658 |
|  | *AtenSHMT1* | 761.847 |
|  | *AtenSHMT3* | 1.44938 |
|  | *AtenSHMT4* | 160.904 |
|  | *AtenSHMT7* | 13.7456 |
|  | *AcarSHMT1* | 393.509 |
|  | *AcarSHMT2* | 8.64246 |
|  | *AcarSHMT4* | 198.397 |
|  | *AcarSHMT7* | 11.4253 |
| *Faveria* | *FcroSHMT1* | 1273.77 |
|  | *FcroSHMT2* | 17.7389 |
|  | *FcroSHMT3* | 40.8527 |
|  | *FcroSHMT4* | 119.281 |
|  | *FcroSHMT7* | 23.5423 |
|  | *FpriSHMT1* | 1303.99 |
|  | *FpriSHMT2* | 12.0737 |
|  | *FpriSHMT3* | 27.2762 |
|  | *FpriSHMT4* | 64.0674 |
|  | *FpriSHMT6* | 3.13479 |
|  | *FpriSHMT7* | 10.9472 |
|  | *FangSHMT1* | 1297.54 |
|  | *FangSHMT2* | 18.0866 |
|  | *FangSHMT3* | 40.77 |
|  | *FangSHMT4* | 89.0073 |
|  | *FangSHMT7* | 31.2286 |
|  | *FpubSHMT1* | 915.567 |
|  | *FpubSHMT2* | 14.5443 |
|  | *FpubSHMT3* | 16.5156 |
|  | *FpubSHMT4* | 114.388 |
|  | *FpubSHMT7* | 34.4853 |
|  | *FbidSHMT1* | 389.849 |
|  | *FbidSHMT2* | 28.1377 |
|  | *FbidSHMT3* | 17.6485 |
|  | *FbidSHMT4* | 138.769 |
|  | *FbidSHMT7* | 8.81943 |
|  | *FkocSHMT1* | 872.881 |
|  | *FkocSHMT2* | 52.6154 |
|  | *FkocSHMT3* | 21.6593 |
|  | *FkocSHMT7* | 30.1597 |
|  | *FtriSHMT1* | 358.804 |
|  | *FtriSHMT2* | 21.7833 |
|  | *FtriSHMT3* | 18.1972 |
|  | *FtriSHMT4* | 95.9287 |
|  | *FtriSHMT7* | 10.2633 |
| *Heliotropium* | *HcalSHMT1* | 1774.94 |
|  | *HcalSHMT2* | 8.7888 |
|  | *HcalSHMT3* | 22.3403 |
|  | *HcalSHMT4* | 51.4802 |
|  | *HcalSHMT7* | 31.1891 |
|  | *HkarSHMT1* | 957.13 |
|  | *HkarSHMT2* | 28.6314 |
|  | *HkarSHMT3* | 20.9451 |
|  | *HkarSHMT4* | 63.4838 |
|  | *HkarSHMT7* | 14.775 |
|  | *HgreSHMT1* | 1494.01 |
|  | *HgreSHMT2* | 17.2131 |
|  | *HgreSHMT3* | 36.092 |
|  | *HgreSHMT4* | 69.9439 |
|  | *HgreSHMT7* | 39.8865 |
|  | *HracSHMT1* | 2554.34 |
|  | *HracSHMT2* | 62.6692 |
|  | *HracSHMT3* | 44.0674 |
|  | *HracSHMT4* | 82.8462 |
|  | *HracSHMT7* | 40.7503 |
|  | *HtenSHMT1* | 448.335 |
|  | *HtenSHMT2* | 27.8604 |
|  | *HtenSHMT3* | 72.7617 |
|  | *HtenSHMT4* | 133.564 |
|  | *HtenSHMT7* | 25.2876 |

Raw data-Table 3 (Continued) Transcriptional Expression Levels of SHMT in 31 Species

| Genus/Tribe | Gene | TPM |
| --- | --- | --- |
| *Heliotropium* | *HtexSHMT1* | 202.382 |
|  | *HtexSHMT2* | 30.593 |
|  | *HtexSHMT3* | 60.1583 |
|  | *HtexSHMT4* | 93.4169 |
|  | *HtexSHMT7* | 28.9573 |
| *Mollugo* | *MpenSHMT2* | 2670.03 |
|  | *MpenSHMT3* | 35.9246 |
|  | *MpenSHMT4* | 99.4063 |
|  | *MpenSHMT7* | 20.3237 |
|  | *MnudSHMT1* | 2757.33 |
|  | *MnudSHMT3* | 58.7042 |
|  | *MnudSHMT4* | 84.1822 |
|  | *MnudSHMT7* | 35.9987 |
|  | *MverSHMT1* | 2505.67 |
|  | *MverSHMT2* | 8.9543 |
|  | *MverSHMT3* | 33.0768 |
|  | *MverSHMT4* | 136.261 |
|  | *MverSHMT7* | 35.6846 |
|  | *McerSHMT1* | 1218.32 |
|  | *McerSHMT3* | 15.6749 |
|  | *McerSHMT4* | 62.4192 |
|  | *McerSHMT6* | 19.2053 |
| *Neurachne* | *NaloSHMT1* | 1433.98 |
|  | *NaloSHMT4* | 61.8091 |
|  | *NaloSHMT7* | 39.2935 |
|  | *NannSHMT1* | 1402.69 |
|  | *NannSHMT4* | 53.0584 |
|  | *NannSHMT7* | 78.1283 |
|  | *NlanSHMT1* | 1548.81 |
|  | *NlanSHMT4* | 74.6054 |
|  | *NlanSHMT7* | 52.205 |
|  | *NminSHMT1* | 1829.42 |
|  | *NminSHMT4* | 21.4609 |
|  | *NminSHMT7* | 115.367 |
|  | *NmueSHMT1* | 268.577 |
|  | *NmueSHMT4* | 75.214 |
|  | *NmueSHMT7* | 60.9327 |
|  | *NmunSHMT1* | 354.89 |
|  | *NmunSHMT4* | 100.661 |
|  | *NmunSHMT7* | 41.2062 |
| Salsoleae | *SjunSHMT1* | 950.504 |
|  | *SjunSHMT2* | 163.2049 |
|  | *SjunSHMT4* | 305.899 |
|  | *SjunSHMT7* | 57.0131 |
|  | *OlarSHMT1* | 1252.49 |
|  | *OlarSHMT2* | 24.0606 |
|  | *OlarSHMT3* | 4.41206 |
|  | *OlarSHMT4* | 359.682 |
|  | *OlarSHMT7* | 43.4084 |
|  | *SfolSHMT1* | 367.696 |
|  | *SfolSHMT2* | 9.16376 |
|  | *SfolSHMT4* | 161.359 |
|  | *SfolSHMT7* | 54.1232 |
|  | *XarbSHMT1* | 555.883 |
|  | *XarbSHMT2* | 14.4538 |
|  | *XarbSHMT3* | 14.9661 |
|  | *XarbSHMT4* | 205.419 |
|  | *XarbSHMT7* | 24.6612 |
|  | | |

Raw data-Table4 Melting temperature (Tm) values of the *SHMT* in different tissues of four Salsoleae species.

| tissue | gene name | melt TM | tissue | gene name | melt TM |
| --- | --- | --- | --- | --- | --- |
| Root | *SjSHMT1* | 82.21317 | Root | *SfSHMT1* | 81.78425 |
|  | *SjSHMT2* | 83.17038 |  | *SfSHMT7* | 83.12162 |
|  | *SjSHMT4* | 82.33957 |  | *SfSHMT4* | 80.78299 |
|  | *SjSHMT7* | 76.54549 |  | *SfSHMT2* | 81.98925 |
|  | *Sjactin* | 80.77735 |  | *Sfactin* | 80.68233 |
| Stem | *SjSHMT1* | 82.16325 | Stem | *SfSHMT1* | 81.335 |
|  | *SjSHMT2* | 83.17085 |  | *SfSHMT7* | 83.02282 |
|  | *SjSHMT4* | 82.11242 |  | *SfSHMT4* | 80.7059 |
|  | *SjSHMT7* | 79.51788 |  | *SfSHMT2* | 81.91548 |
|  | *Sjactin* | 80.52591 |  | *Sfactin* | 80.55492 |
| Leaf | *SjSHMT1* | 81.98647 | Leaf | *SfSHMT1* | 81.26116 |
|  | *SjSHMT2* | 83.09481 |  | *SfSHMT7* | 82.97065 |
|  | *SjSHMT4* | 82.21363 |  | *SfSHMT4* | 80.70749 |
|  | *SjSHMT7* | 79.54351 |  | *SfSHMT2* | 81.91376 |
|  | *Sjactin* | 80.39951 |  | *Sfactin* | 80.60682 |
| Root | *OlSHMT4* | 81.49792 | Root | *XaSHMT2* | 82.31773 |
|  | *OlSHMT2* | 81.79461 |  | *XaSHMT1* | 83.70206 |
|  | *OlSHMT1* | 83.59559 |  | *XaSHMT3* | 80.6568 |
|  | *OlSHMT7* | 81.7197 |  | *XaSHMT7* | 80.27953 |
|  | *OlSHMT3* | 81.5229 |  | *XaSHMT4* | 83.39985 |
|  | *Olactin* | 80.73207 |  | *Xactin* | 80.26285 |
| Stem | *OlSHMT4* | 81.37013 | Stem | *XaSHMT2* | 82.34311 |
|  | *OlSHMT2* | 81.59781 |  | *XaSHMT1* | 83.47535 |
|  | *OlSHMT1* | 83.54247 |  | *XaSHMT3* | 80.58152 |
|  | *OlSHMT7* | 81.5229 |  | *XaSHMT7* | 80.25415 |
|  | *OlSHMT3* | 81.49496 |  | *XaSHMT4* | 83.34975 |
|  | *Olactin* | 77.12803 |  | *Xactin* | 80.01314 |
| Leaf | *OlSHMT4* | 81.32312 | Leaf | *XaSHMT2* | 82.24224 |
|  | *OlSHMT2* | 81.61983 |  | *XaSHMT1* | 83.50074 |
|  | *OlSHMT1* | 83.54565 |  | *XaSHMT3* | 80.55614 |
|  | *OlSHMT7* | 81.59486 |  | *XaSHMT7* | 80.32986 |
|  | *OlSHMT3* | 81.62279 |  | *XaSHMT4* | 83.29919 |
|  | *Olactin* | 77.1283 |  | *Xactin* | 79.93312 |
