# Supplementary material for: Genome-wide identification of SHMT family genes in C3, C3-C4, and C4 Salsoleae s.l. species
Source: PeerJ. 2025 Sep 3;13:e19978. doi: 10.7717/peerj.19978 (PMC12422262; doi:10.7717/peerj.19978)
Supplement: Supplemental Information 1 [file peerj-13-19978-s001.zip › Supplemental Raw Data/SHMT protein sequences.docx]

>SjSHMT1

MAMAMALRKLSNSVNKKPIRNAFNAAPLYFMSSEAALKEKQVSVTWPKQLNAPLEEVDPE

IADIIEHEKTRQWKGLELIPSENFTSVSVMQAVGSVMTNKYSEGYPGARYYGGNEYIDMA

ETLCQKRALEAFRLDPEKWGVNVQPLSGSPANFHVYTALLKPHERIMALDLPHGGHLSHG

YQTDTKKISAVSIFFETMPYRLDESTGYIDYDQKTEEVDILVWLLCFPRYNWSILLQMEK

SAALFRPKLIVAGASAYARLYDYERIRKVCDKQKAIMLADMAHISGLVAAGVIPSPFDYA

DVVTTTTHKSLRGPRGAMIFFRKGVKEINKQGKEVLYDYEDRINQAVFPGLQGGPHNHTI

TALAVALKQAVTPEYRAYQEQVLSNCSKFAEALSGMGYELVSGGTDNHLVLVNLKNKGID

GSRVEKIMEAVHIAANKNTVPGDVSAMVPGGIRMGTPALTSRGFIEEDFVKVAEYFDAAV

KLALKIKAATKGTKLKDFVATFQSGAFDSDIAKLRGEVEEYAKQFPTIGFEKETMKYKN

>SjSHMT2

MAISMAVRRLSSSSIRPRFNVGSHYYMSSLSNEAGINNRSRVNWINQLNEPLEVIDPEIA

DIIELEKARQWKGLELIPSENFTSASVMQAVGSVMTNKYSEGYPGARYYGGNEYIDMAES

LCQKRALEAFQLDPEKWGVNVQSLSGSPANFQAYTALLKPHERIMALDLPHGGHLSHGYQ

TDTKKISAVSIFFETMPYRLDESTGYIDYDQLEKSAVLFRPKLIVAGASAYARLYDYARI

RKVCDKQKAILLADMAHISGLVAAGVIPSPFEYADIVTTTTHKSLRGPRGAMIFFRKGVK

EINKKGQEVRYDFEDKINQAVFPGLQGGPHNHTITGLAVALKQAMTPEYKAYQQQVLSNC

TAFAQKLAENGYNLVSGGTDNHLVLVNLRDKGIDGSRVEKVLEAVHIASNKNTVPGDVSA

MIPGGIRMGTPALTSRGFMEEDFAEVADFFDAAVKLALKIKADTQGTKLKDFVATMQSSN

NFQSEIIKLRQEVEDYAKQFPTIGFEKESMKYKD

>SjSHMT4

MQVQQISSSSTSVYNSRGTIVFSQAFNSPFKSRPHFIKHLVCNTSSSSFSLSISPKNTHF

SLLSPPKSAMEPVNSWGNTSLEEIDPEIHDLIEKEKRRQCRGIELIASENFTSFAVIEAL

GSALTNKYSEGMPGNRYYGGNEHIDVIENLCRSRALECYRLNPEKWGVNVQPYSGSPANF

AAYTAVLNPHDRIMGLDLPSGGHLTHGYYTAGGKKISATSIYFESLPYKVNSQTGYIDYD

KLEEKALDFRPKLIICGGSAYPRDWDYARFRAIADKCGALLLCDMAHISGLVAAQEAASP

FEYCDIVTTTTHKSLRGPRAGMIFYRKGPKPPKKGQPEDAVYDFEDRINFAVFPSLQGGP

HNHQIGALAVALKQAMSPGFKAYAKQVRANAVAIGNYLMSKGYSIVTGGTENHLVLWDLR

PLGLTGNKVEKLCDLCNITVNKNAVFGDSSALAPGGVRVGAPAMTSRGLVEKDFEQIGEF

LHRAVALTLSIQKEHGKLLKDFNKGLVNNKEIEQLKADVEKFASSYDMPGFLKSELKYKD

>SjSHMT7

MDLTPSQSNLSLGFLSHATANPPNPNRISINDDSISFQIDSRLRESTNPISSIPLQLLEQ

TAQNCRKMNENSRSIDDKDRVSENTKDTDGADERGAVNGDEEVEEFRILGHSMCLKRRRD

REDSVSSSSADSAKRMFFDSSLELRRCAVRSWGNQSLRSADPEIYDIMEREKLRQCKGIE

LIASENFVCKAVMEALGSFLTNKYSEGMPGQRHYGGNQYIDEIETICCERALAAFDLSPD

DWGVNVQPYSCTSANFAVYTALLLPGDRIMGLDILSGGNTSHGCYTPNGRKVSGASIFFE

TLPYKVNPQTGYVEYDKLEEKALDFHPKILICGGSSYPREWDYARLRQIADKCGAVLMCD

MAQISGLIAAKECVSPFEYCDIVTSTTHKSLRGPRGGIIFFRKGKKPRKRSMLLNPSDDS

ELYDFEEKINFAVFPALQGGPHNNHIAALAIALKQVATPVYKAYMQQVKKNAQALASALL

RRKCRLITGGTDNHLLLWDLRPLGLTGKNFEKVCETCYISLNKVALFGDNGSITPAGVRI

GTPAMTTRGLLESDFEVIADFLIRAAHITSKVQREHGKLSECFLKGLQSNDDIVELRTRI

EVFAAQFSMPGFDDV

>OlSHMT1

MALAMALRKLSNSVNKKPIRNAFNGAPLYFMSSEAALKEKQVSVTWPKQLNAPLEEVDLE

IADIIEHEKTRQWKGLELIPSENFTSVSVMQAVGSVMTNKYSEGYPGARYYGGNEYIDMA

ETLCQKRALEAFRLDPEKWGVNVQPLSGSPANFHVYTALLKPHERIMALDLPHGGHLSHG

YQTDTKKISAVSIFFETMPYRLDESTGYIDYDQLEKSAALFRPKLIVAGASAYARLYDYE

RIRKVCDKQKAIMLADMAHISGLVAAGVIPSPFDYADVVTTTTHKSLRGPRGAMIFFRKG

VKEINKQGKEVLYDYEDRINQAVFPGLQGGPHNHTITALAVALKQAVTPEYRAYQEQVLS

NCSKFAEALSGMGYELVSGGTDNHLVLVNLKNKGIDGSRVEKIMEAVHIAANKNTVPGDV

SAMVPGGIRMGTPALTSRGFIEEDFVKVAEYFDAAVKLALKIKAATKGTKLKDFVATFQS

GAFDSDIAKLRGEVEEYAKQFPTIGFEKETMKYKN

>OlSHMT2

MAMSMAVRRLSSSSSIRPRFNVGSHYYMSSLSNEAGINNRSRVNWINQLNESLEVIDPEI

ADIIELEKARQWKGLELIPSENFTSASVMQAVGSVMTNKYSEGYPGARYYGGNEYIDMAE

SLCQKRALEAFQLDPEKWGVNVQSLSGSPANFQAYTALLKPHERIMALDLPHGGHLSHGY

QTDTKKISAVSIFFETMPYRLDESTGYIDYDQLEKSAVLFRPKLIVAGASAYARLYDYAR

IRKVCDKQKAILLADMAHISGLVAAGVIPSPFEYADIVTTTTHKSLRGPRGAMIFFRKGV

KEINKKGQEVRYDFEDKINQTVFPGLQGGPHNHTIAGLAVALKQAMTPEYKAYQQQVLSN

CTAFAQKLADNGYNLVSGGTDNHLVLVNLRDKGIDGSRVEKVLEAVHIASNKNTVPGDVS

AMIPGGIRMGTPALTSRGFMEEDFAKVADFFDAAVKLALKIKADTQGTKLKDFVATMQSS

NNFQSEIIKLRQEVEDYAKQFPTIGFEKKSMKYKD

>OlSHMT3

MQVCSGASNIAAMNLNMIAFGYHQCRSKIMSPVSFTNKSSSLFEYDYDYSKKASSRLLCV

SQSHSNSNSNSLVTSRPPSSVSLPSPDTDTHYPGDQSSFKDNEPSDADPQVYNIIGKEKE

RQFKSLELIASENFTSRAVMEAVGSCLTNKYSEGLPGKRYYGGNEYIDELETLCQQKALE

AFHWDGQIWGVNVQPLSGSPANFEVYTALLNPHDRIMGLDLPHGGHLSHEGLVDYDMLEK

TATLFRPKLIIAGASAYPRDFDYPHMRKIADSVGSFLMMDMAHISGLVAASVSLRGPRGG

MIFYKKDTILGVDLESAINNAVFPGLQGGPHNHTIGGLAVCLKELMGARVERILEMASIT

LNKNSLPGDKSALVPGGIRIGSPAMTTRGFKESEFVATAELIHEGVQIALEANKLMPKTK

LHEFLKYVGSPEFPLRDRVLELQRRVEVVTTQFPLPGL

>OlSHMT4

MQVHQISPSSSAVYNFKGTKVFSQPLNSPFKSPPHFIKHLVCNSSSSSSSLSITPKNTHF

SLLSPPKSAMEPVNSWGNTSLEEIDPEIHDLIEKEKRRQCRGIELIASENFTSFAVIEAL

GSALTNKYSEGMPGNRYYGGNEHIDVIENLCRSRALECYRLSPEQWGVNVQPYSGSPANF

AAYTAVLNPHDRIMGLDLPSGGHLTHGYYTAGGKKISATSIYFESLPYKVNSQTGYIDYD

KLEEKALDFRPKLIICGGSAYPRDWDYARFRAIADKCGALLLCDMAHISGLVAAQEAASP

FEYCDIVTTTTHKSLRGPRAGMIFYRKGPKPPKKGQPEDAVYDFEDRINFAVFPSLQGGP

HNHQIGALAVALKQAMSPGFKAYAKQVRANAVAIGNYLMSKGYSIVTGGTENHLVLWDLR

PLGLTGNKVEKLCDLCNITVNKNAVFGDSSALAPGGVRVGAPAMTSRGLVEKDFEQIGEF

LHRAVTLTLSIQKEHGKLLKDFNKGLVNNKEIEQLKADVEKFASSYDMPGFLKSELKYKD

>OlSHMT7

MDLTPSQSNLSLGFLSHATANPPNPNRISINDNSISFQIDSRLCESSNPISAIPLQLLEQ

AAQNCRKMNENSRTIDDKDTVWENTKDTDGADERGAVNGDEEVEEFRILGHSMCLKRRRD

REDSVSSSSADSAKRVFFDSSLELRRCAVRSWGNQSLRSADPEIYDIMEREKLRQCKGIE

LIASENFVCKAVMEALGSFLTNKYSEGMPGQRHYGGNQYIDEIETMCCERALAAFDLSPD

DWGVNVQPYSCTSANFAVYTALLLPGDRIMGLDILSGGNTSHGCYTPNGRKVSGASIFFE

TLPYKVNPQTGYVEYDKLEEKALDFHPKILICGGSSYPREWDYARLRQIADKCGAVLMCD

MAQISGLIAAKECVSPFEYCDIVTSTTHKSLRGPRGGIIFFRKGKKPRKRSMLLNPSDDS

ELYDFEEKINFAVFPALQGGPHNNHIAALAIALKQVATPVYKAYMQQVKKNAQALASALL

RRKCRLITGGTDNHLLLWDLRPLGLTGKNFEKVCETCYISLNKVALFGDNGSITPAGVRI

GTPAMTTRGCLESDFEVIADFLIRAAHITSKVQREHGKLSECFLKGLQSNDDIVELRTRI

EVFAAQFSMPGFDDV

>SfSHMT1

MAMAMALRKLSNSANKNPIRNALNAAPVYFMSSEAALKEKQISVTWPKQLNAPLEEVDPE

IADIIEHEKTRQWKGLELIPSENFTSVSVMQAVGSIMTNKYSEGYPGARYYGGNEFIDMA

ETLCQKRALEAFRLDPEKWGVNVQPLSGSPANFHVYTALLKPHERIMALDLPHGGHLSHG

YQTDTKKISAVSIFFETMPYRLDESTGYIDYDQMEKSAVLFRPKLIVAGASAYARLYDYE

RIRKVCDKQKAIMLADMAHISGLVAAGVIPSPFDYADVVTTTTHKSLRGPRGAMIFFRKG

VKEINKLGKEVLYDYEDRINQAVFPGLQGGPHNHTITALAVALKQAVTPEYRAYQEQVLS

NSSKFAEALSGMGYELVSGGTDNHLVLVNLKNKNIDGSRVEKIMEAVHIAANKNTVPGDV

SAMVPGGIRMGTPALTSRGFIEEDFVKVAEYFDAAVKLALKIKAATKGTKLKDFVATFQS

GSFDSDIAKLRDEVEEYAKQFPTIGFEKESMKYKN

>SfSHMT2

MAMSMAVRRLSSSSSSIRPRFNVASHYYMSSLSNEAGINNRSRVNWINQLNEPLEVIDPE

IADIIELEKARQWKGLELIPSENFTSASVMQAVGSVMTNKYSEGYPGARYYGGNEYIDMA

ESLCQKRALEAFQLDPEKWGVNVQSLSGSPANFQAYTALLKPHERIMALDLPHGGHLSHG

YQTDTKKISAVSIFFETMPYRLDESTGYIDYDQLEKSAVLFRPKLIVAGASAYARLYDYA

RIRKVCDKQKAILLADMAHISGLVAAGVIPSPFEYADIVTTTTHKSLRGPRGAMIFFRKG

VKEINKKGQEVSYDFEDKINQAVFPGLQGGPHNHTIAGLAVALKQAMTPEYKAYQQQVLS

NCTAFAKKLADNGYNLVSGGTDNHLVLVNLRDKGIDGSRVEKVLEAVHIASNKNTVPGDV

SAMIPGGIRMGTPALTSRGFMEEDFAEVADFFDAAVKLALKIKADTQGTKLKDFVATMQS

SNNFQSEIIKLRQEVEDYAKQFPTIGFEKESMKYKE

>SfSHMT4

MQVHQISSSSATLYNFKGRKVFSQPSNSPFKSPPHFIEHLVCNSSSSSFSLSITPKNTHF

SLLSPPKSAMEPVSSWGNTSLEEIDPEIHDLIEKEKRRQCRGIELIASENFTSFAVIEAL

GSALTNKYSEGMPGNRYYGGNEHIDVIENLCRSRALECYRLSPEKWGVNVQPYSGSPANF

AAYTAVLNPHDRIMGLDLPSGGHLTHGYYTAGGKKISATSIYFESLPYKVNSQTGYIDYD

KLEEKALDFRPKLIICGGSAYPRDWDYARFRAIADKCGALLLCDMAHISGLVAAQEAASP

FEYCDIVTTTTHKSLRGPRAGMIFYRKGPKPPKKGQPEDAVYDFEDRINFAVFPSLQGGP

HNHQIGALAVALKQAMSPGFKAYAKQVRANAVAIGNYLMSKGYSIVTGGTENHLVLWDLR

PLGLTGNKVEKLCDLCNITVNKNAVFGDSSALAPGGVRVGAPAMTSRGLVEKDFEQIGEF

LHRAVTLTLSIQKEHGKLLKDFNKGLVNNKEIEQLKADVEKFASSYDMPGFLKSELKYKD

>SfSHMT7

MDLTPSQSNLSLGFLSHATANPPNPNRISINDDSISFQIDSRLRESSNPISSIPLQLLEQ

TAQNCRKMNENSRSIEDKDRVSENTKDTEGGDERGAVIGDEEVEEFRILGHPMCLKRRRD

REDSVSSSSADSAKLMFFDSSLELRRCAVRSWGNQSLRSADPEIYDIMEREKLRQCKGIE

LIASENFVCKAVMEALGSFLTNKYSEGMPGQRHYGGNQYIDEIETMCCERALAAFDLSPD

DWGVNVQPYSCTSANFAVYTALLLPGDRIMGLDILSGGNTSHGCYTPSGRKVSGASIFFE

TLPYKVNPQTGYVEYDKLEEKALDFHPKILICGGSSYPREWDYARLRQIADKCGAVLMCD

MAQISGLIAAKECVSPFEYCDIVTSTTHKSLRGPRGGIIFFRKGKRPRKRSLLLNASDDS

ELYDFEEKINFAVFPALQGGPHNNHIAALAIALKQVATPVYKAYMQQVKKNAQALASALL

RRKCRLITGGTDNHLLLWDLRPLGLTGKNFEKVCETCHISLNKVALFGDNGSITPAGVRI

GTPAMTTRGCLESDFEVIADFLIRAAHITSKLQREHGKLSECFLKGLQSNDDIVELRTRI

EVFAAQFSMPGFDDV

>XaSHMT1

MAMAMALRKLSNSVNKKPIFNGAPLYFMSSEAALNEKQVSVTWPKQLNAPLEEVDPEIAD

IIEHEKTRQWKGLELIPSENFTSVSVMQAVGSVMTNKYSEGYPGARYYGGNEFIDMAETL

CQKRALEAFRLDPEKWGVNVQPLSGSPANFHVYTALLKPHERIMALDLPHGGHLSHGYQT

DTKKISAVSIFFETMPYRLDESTGYIDYDQMEKSATLFRPKLIVAGASAYARLYDYERIR

KVCDKQKSIMLADMAHISGLVAAGVVPSPFDYADVVTTTTHKSLRGPRGAMIFFRKGVKE

INKQGKEVLYDYEDKINQAVFPGLQGGPHNHTITALAVALKQAMTPEYRAYQEQVLSNCT

KFAEALSKLGYELVSGGTDNHLVLVNLKNKGIDGSRVEKIMEAVHIAANKNTVPGDVSAM

VPGGIRMGTPALTSRGFIEEDFAKVAEYFDAAVKLALKIKAETKGTKLKDFVATFQSGAF

DSDIAKLRNEVEEYAKQFPTIGFEKETMKYKN

>XaSHMT2

MAMSMAVRRLSSSSSPSIRPRFNVGSHYYMSSLSNEAGIKNRSSVNWTNQLNEPLEVIDP

EIADIIELEKARQWKGLELIPSENFTSASVMQAVGSVMTNKYSEGYPGARYYGGNEYIDM

AESLCQKRALEVFQLDPEKWGVNVQSLSGSPANFQAYTALLKPHERIMALDLPHGGHLSH

GYQTDTKKISAVSIFFETMPYRLDESTGYIDYDQLEKSAVLFRPKLIVAGASAYARLYDY

ARIRKVCDKQKAILLADMAHISGLVAAGVIPSPFEYADIVTTTTHKSLRGPRGAMIFFRK

GVKEINKKGEEVRYDFEDKINQAVFPGLQGGPHNHTIAGLAVALKQAMTPEYKAYQEQVL

SNCTAFAQKLADNGYNLVSGGTDNHLVLVNLRDKGIDGSRVEKVLEAVHIASNKNTVPGD

VSAMIPGGIRMGTPALTSRGFMEEDFAKVADFFDAAVKLALKIKADTKGTKLKDFVATMQ

SNDNFQSEIKKLRQEVEDYAKQFPTIGFEKESMKYKD

>XaSHMT3

MQACSGASSAATMTMNMMACECRSPVSLTTKLSLFGYDHSNKASFRLSCASHTNSLVTSR

PPSSASLPSPDTDYLDDQSGFKDNELSDADPQVYDIIGKEKERQFKSLELIASENFTSRA

VMEAVGSCLTNKYSEGLPGKRYYGGNEYIDELETLCQQRALEAFRLDGQSWGVNVQPLSG

SPANFEVYTALLNPHDRIMGLDLPHGGHLSHGFMTPKRRVSGTSIYFESMPYRLDESTGL

VDYDMLEKTATLFRPKLIIAGASAYPRDFDYPRMRKIADAVGAFLMMDMAHISGLVAASV

VGNPFEYCDVVTTTTHKSLRGPRGGMIFYKKDTILGVDLESAINNAVFPGLQGGPHNHTI

GGLAVCLKYAQSQEFKAYQSNVVANCRALAQRLVELEYKLVSGGSDNHLVLVDLRPLGID

GARVERILEMASITLNKNSVPGDKSALVPGGIRIGSPAMTTRGFRESEFVATAEFIHEGV

QIALEANKLIPKTKLQEYLKYVGSPEFPLRDRISDLRRRVEVVTTQFPLPGL

>XaSHMT4

MQVQQISSATTAVYNTKGRRVISQPLKSPFKSAPHFIKHLVCNSSKPSISLSINTPKKTQ

ICNLSPPKSAMEAVKTWGNTSLEEIDPEIHDLIEKEKRRQCRGIELIASENFTSFAVIEA

LGSALTNKYSEGMPGNRYYGGNEHIDVIENLCRSRALECYRLNPEQWGVNVQPYSGSPAN

FAAYTAVLNPHDRIMGLDLPSGGHLTHGYYTAGGKKISATSIYFESLPYKVNSQTGYIDY

DKLEEKALDFRPKLIICGGSAYPRDWDYARFRAIADKCGALLLCDMAHISGLVAAQEAAS

PFEYCDIVTTTTHKSLRGPRAGMIFYRKGPKPPKKGQPEDAVYDFEDRINFAVFPSLQGG

PHNHQIGALAVALKQAMSPGFKAYAKQVRANAVAIGNYLMSKGYSIVTGGTENHLVLWDL

RPLGLTGNKVEKLCDLCNITVNKNAVFGDSSALAPGGVRVGAPAMTSRGLVEKDFEQIGE

FLHRAVTLTLSIQKEYGKLLKDFNKGLVNNKEIEQLKADVEKFASSYDMPGFLKSELKYK

D

>XaSHMT7

MDLTPSQSNLSLGFLSHATANPPNPNRISINDDSISFQIDSRLRESSNPISSIPLQLLEQ

TAQNCRKMKENSRSVDDRDRVSENTKDTDGAEEREAGNGDEEVEEFRILGHPMCLKRRRD

REDSVSSSSVDSTKRMLLDSSLEVRRCAVRSWGNQSLRCADPEIYDLMEREKLRQCKGIE

LIASENFVCKAVMEALGSFLTNKYSEGMPGQRHYGGNQYIDEIETMCCQRALAAFDLSPD

HWGVNVQPYSCTSANFAVYTALLLPGDRIMGLDILSGGNTSHGCYTPNGRKVSGASIFFE

TLSYKVNPHTGYVEYDKLEEKALDFHPKILICGASSYPRDWDYARLRQIADKCGAVLMCD

MAQISGLIAAKECVSPFEYCDIVTSTTHKSLRGPRGGIIFFRKGKKPRKRSMLLNPSDDS

ELYDFEEKINFAVFPALQGGPHNNHIAALAIALKQVATPVYKAYMQQVKKNAQALASALL

RRKCRLITGGTDNHLLLWDLRPLGLTGKNFEKVCETCHISLNKVALFGDNGSITPAGVRI

GTPAMTTRGCLESDFEVIADFLVQAAHITSKVQREHGKLSECFLKGLQSNDDIVELRTRI

ETFAAQFSMPGFDDV
