# Supplementary material for: Genome-wide identification of SHMT family genes in C3, C3-C4, and C4 Salsoleae s.l. species
Source: PeerJ. 2025 Sep 3;13:e19978. doi: 10.7717/peerj.19978 (PMC12422262; doi:10.7717/peerj.19978)
Supplement: Supplemental Information 1 [file peerj-13-19978-s001.zip › Supplemental Raw Data/SHMT1 2-kb upstream promoter sequences from four species.docx]

>SjSHM1

tatatgtttagataagttcaattcaggtttagtaaggtctgatcgagtttaacacaacacccaaaaataaatttcaatgaggatctatcagggtcaaataagcctaataaattcagttcacgtaaattttcaggtgaagtaaacacaccctaagctgttgtacaaaataataaatcgtgcattttaaacatgtttttgataatattttaaatttttttgttatgttaattaaataataaattcagttaaatgttttttttaaagaaaatttagatatcactcgtgcatcgcacaaagtat

aaccctagtaaaataaaattaggatatcacactccattttttattgttattccctctttcctaggccattctaaattgttaatccctctttcactctttcactctatttcttattgcttttttttcctaccttcttttaccccacttgtaaaaccaattgactattctactttctaaaaactccttaatatttgtaatatttgcttttttggagtcaaattcttataactttgactattaattcccatcaatatatatgttaaaactctatcatgagatcttgtttaattcgtcatagcatgtagtttccaaatatgtaaatttgataaattttataaacacgaaatcaaagatatttatggtcaaaagtgcgtcttgaaaactgtgaaaaagcagatgttgcaaatatttagaaattgagggggaggggtgataaatcccgaacaagagttgggcatttatgcccccatattacctatacaagaggtgtctatgccatacaaaaggtgtccatgtttgttttttttccttcggctatccttttcatgtggatctcagcattaaagttaaattttttattggaaaacctataaagataacacttttagaaaatagacacctttttcagaaaataacagacactttttttaaatggtatagacaacttgttattggcctaaggatgtaactggggcgaggtgggacgaatgttggcattcccatctccatccccgcatcactttttcatcttcatccccgcagtgggtccgaaactcatcccatccccgccccaccatgtacaaaattagaacaatcctcgtcccaccacccacttagatatccgtatccattcccaccaaatccccaccttccccgccccaccgtccacttaatcctcatttgaaactcactaaataatactcatataccatttaaaatttcaaatacgaataataaaaaaaatatacaaatggaaaaaggttataaaaaaaaagttataaacttgtaattagttatgggaaaattttaatttttaacaccagatttgttgaaatttcttattttttagtggggatgaagcggggcgggtggggaaggggtggatacaacattaaatccacccccgccccatcactcatggcaggtatgactttcatccccatcctcacccgccaaactagggtggggatgatgcggttctcctacttgacccgccccacttacatagttacataattacattcctatattgaccagacttgcccaactcgtattctgggttaatattttttttttttttagtactctagctttgattaaacaagcatgataaatgactcacttgcatgcacactctctttggtgtgggagagtataagatatggaccatttgagcacagttggtggctaagagttcctggggacaatatgtgtggtaggtgaaacacaaacataactttaatttggaaacaaacaagataaactcaatctaaataatggagtagtgatggagcaacaaaaacaaaacaaaacaaatactcatagtgcgccacaaataaactaatttttcaaaaagtcgttgttttattaacaaccacttatacctcacccaaatcactcaccacacttcacaccccaaaaacagtgagtcatttgagtgagtgataacagagaaagaagtaagagagaaaagaaaaact

>OlSHM1

aatgaaaggagctgtatgctaagctgcaacaaggaggttcgagatataacgttgtggaaggcccctcctaatggtgtattaaaaattaatactgatgctgcagtgtttaaggagaagggagtgggtatgggggctattgttagagataattttggtaatgtagttagggcggcttgcaagcatgcagatgaatcaaccgttggatgttgatatatatagcagaagcgaaggctgttgctcttggtttacagttggcggttcagactggaggaagcagcattatggtggagagtgactctttgagggtcattaattcaattattaatacagtgatggaaaactccgatcctattttggtcttgtaattagagaggttcttgatattctaaatgctttgagtctattagttttaatcatgttagagagtcaaacccgttgatttctccacaagaatttgggttggtgattgcccttgtgagattgaggatgtagtagcttttaatgcttgtaacaactcttattcttaataatatttatcttttcccatcaaaaaaagtaaaataaaataaaatatttcatccgtctatctttagttgccagatgtgccacatttgaatatcttgagctttatataatgaaaaaattacttaattggaaagtacacattgagacaaatcaaccaaaatctcatataactatgttatgtcttatgtatctaaattcgaagtgagtcaaaattcaatgtgaatattgcttcaaatcctaatgtaaaaaatattccatctgtctatctttagttgccagacttgccacatttgaatatcttgagctttatataatgaaaaaattacttaattggaaagtacacattgagacaaatcaaccaaaatctcatataactatgttatatcttatgtatctaaattcgaagtgagtcaaaattcaatgtgaatattgcttcaaatcctaatgtagcaatttaaaagaacacaaaggaagtaatattcaagtaggatatcacattccctttcatattatttttccctttctcctcacatgtcattctaagttcatattcttttgagtttctcactttattttttagtaatttgcaacaactactgtcaaacgggcttgggttgcacccgccatggggaagtgcaaatccactttacaagaccaaggagattctacctcaaaaccatatagcaataaggggagtaactctagccttataaagtggatacaacttacttatttcatcaatgtgggattcctcaccataacactccctcttaattccaaactcaataatgtgggacagcacatacactcacatgtgggttgtttatgggctcaacactccccttcacatgtgagttgtccataattatggcccagatcataacaatcccaacctgggctctgataccatgtcaaacgggtgtgcccgccactgaggagtgcaaatccactttaacatggtatcggaaccaacatcacatatccgaatcttctcaccccttccttccaagtggaattaattcgtgtcggcatgaaggggcttgtgctgcatctacatgtcaaactcaaatggcctttcatgtgacgagaagtgacataatattaatattaattctgacttagcatttggttgaagagatccactaacaactctgtccgctgtgggagtataaggtatggaccgtttgagcacagttggtggctaagagttcctagggacagaaatgtgtggtaggtgaaacgcacaaacataacttcacttgcaaacaaacaagataaactcaatctaaatctatataatggagtgatggagcaacaaaaacaaaagaaaatctcaaagtgcgccacaaaaaacactaaattttcaaaaagtcgttgttttattaacaaccacttatacctcacccaaatcactcaccccacttcacaccccaaaacagtgagtgataacagagaaaaattgagagagaaaaact

>SfSHM1

agaccccagtttgcatgtctcctcttcatctggctcaacctcttcgttacctttatctagcccagaatcagccaccattgttgcaaatttcttctggaccttatccatttcgtctttgtaacgacacttaaaccatttgagtggtccacgacagagaagacaggtcaagggtcgtggtttgttgttgcccgatgaagaaccggcatgggtttttgtagttgttggaggaggaccctcctctctgagaattatctccccaacttcggcccttattgcccacgctgtttgagccactttaggaattgcttgttgagggctttgacgtgatgatagtcggactattgtttcccttcttttgattttaattccttggagtagagtagtccatcaacctctcggacgcagtgatagcggtagacagtgtatcaactttctggcgccataactctctttgggcccagtctttcaacccttgtacaaagttgaacacacgatccttctctgccatgtccttaacatccagcatgcatgtcgagtattccttaacatagtctcggatagatccagtgtgttgcagtgttttgagcttttggcgggcaacaaactcggtattctctgggtagtattgctcgatgaggttcttcttgaactcggcccacgtatcaattttcacctttccggcctgcatgtcggcatgttttgtccgccaccagagcttggcgtcatcaaccaaatacatcgaagcagtgacgaccttgatattctcatccatatggcaaatctcgaaatactgctccatgtcaaatgtgaaattgtctacctctattgcgtctcgtgcacccccgtattcttttatactgggtggcttcacctttgttgaagcaacgtttggggtgtctttactcaatgcccgcatcagggtagcgtgcttgtccatcaattcctccacttgaccttggacaaagtttagttgttcttgtatcccgtctttctcggtgaaaagaacattcaattgctcttgcatcgcatctctttcagtggtcaggtcatggatctcggtctcacgctgatcaatctgttttgcctgacctatcactatttcttctagctgggtccattgctcggacttgccctcaagccaggcaatccgttctctatctgttagttctgccatcgtgtataattttgcacacgatttcttctcagggtctcttacagcttaatgttttgtaacctggctctgataccaactgtcacggtccgagtgtttcgacaccggaacgtgcgacgcacccttgcttatttgcgagcaagaatgcaagcctgaaggggaaagactcatgagcacacaacaacacaagtagagttcgaggagttcgagttgacaggtgtttgggacttacgcgggcgactaggccaaaccgggcgcaagagggttcgaacacctagtgtgttgtctaggcaacaaaagaatttgaaggttctaatgcaacgcaggctcaatatctataaaggccaattaacaacacgaaaacattaaagcaagacaacacggatagggagtaatgaacttggtttcattcatacccctaaagaggttaaattacgatttacaaaattcccccatgagaccctcaaaggggagcaactccatacaatataaagcttagaaaataaactaagtaaagcatagaaaaggacctaagtacaagctactaggctaatgtgggaggattctaacacctagagacagaatttgtggtaggtgtaagtgtaacaaataaacgtaactttatttgcaaacaaacaagataactcaatctaaataatggagttatggggcaacaaaaacaaaagaaaatctgatagtgcgccacagataaactgaattttcaaaatgtcgttgtttcattaacaaccacttatacctcacccaaattactcaccacagttcacacccccataacagtgactccttctttcgtgagagaaaaaacacc

>XaSHM1

caataaacaagcttgaacaaaaatttaaagctcgttaaataaccgagtcaagctcaaacaaccttatgtttaacttgtgaatgtttgtgaacaactcgattaaagctcattcaactatacaaaactgaaatttaaatattgtttaactatctctttatatcttagaatttagagattataaacggctttggtacgcatgtgaataaatttgatcgttgctaccgtttggagaagtgaagatttttttattttttttttttcaatttaatggttatataaaaactctaaaatcattagccttgcctaaaatgcaactcaaggcttgtttatttagccaagctcaagttaaagctcgattaagatcaagctcgaacttgaaaagcaaatgagtgcgtttgtgagcaaagctcgaaccagaattttaaaagcttgttcgagttcatgatagtttaatcactaataatttcattcaagcttagctcgagcagcctaaaactcgacttaactaatttgcagctcttatttttagaggagtacaataaatgactcacttgcagtactctcttgactgggaagtacctttccctgatcgtcgacaaaaaactttagcacatgcattggggcacactttcataacaatatatgacttgggcaccatgtgcatttaatagtaggtagactccatggaaatcgcacggacaagataaatcacgcacagatgaaattgaattataaatagatttagacatgggcattcggccgggtagcacgcaggccatacgggcacggcacgagttgggcccggcccagcacgggcccaccattggcacggcacggcacgagaggcacgctcatgggccgtgggccgtgtatggacaacattttcaaaattgggcacgagcccagcacgagcacgacgggccggcccgcccattggcacactcattttgaacaaatattaataaaaaatagaaattgtaactaaggcacgaggcacggcacgtgtcggcccggcacgaaacgtgggtcgggttgggcgtgggcctttcttattgaaaattggcacgatcccagcacggcacgacattgacttggcccgtttaggcacggcccgccatggcacgaggcccgtgggcttggcacgtgggccgtcccggcacggcccatgcccacctttaaatagattacatatggaaaaacatagacgagatatgaaaaaaacataaactactactctaaattacaaaaaacattgttgttcattggataagtcattagaaatgtgagccccaccttctaaaaagctgttggcggcactattttatagattctacatctccaaaaaaatcaaggtagtggggcaaaaattacactcttacccttgttatatgctttttttggtaaccctctcttggtgatgggtttatcacccctcctcttttgggcgtgacgtgagagtataaggccctgttctttccagcttcttttaagttcagttcggctgtgagaagttcagttcagttcagttcagctgtgagaatttcatttcagttcagctgtgcttagttcatttttacactacatttcagttcagttcagttccgttcagctgtaagaagttcagttcagttcagttcagctgtgagaatttcattcagttcagttcagcaaatttcagatgaaaaaaacagggcctaagatatatatgggccgtttgagcacagttggtggctacgagtccctggggacagaatgtgtggtaggtgaaacacaaacataactttagttacaaacaaacaagtaaacaacatacctcaatccaaatccaaataatggaatgatggagcaaaaaaatacaaaagaaaatctcaaagtgcgccacaagaaaactaaaatttaaaaaagtccgttgttttattataaaccacttatacctcaccctaaatcattcaccacacttgattcccaaaagagtgattggagtgatcgagagataaact
