# Supplementary material for: Genome-wide identification of SHMT family genes in C3, C3-C4, and C4 Salsoleae s.l. species
Source: PeerJ. 2025 Sep 3;13:e19978. doi: 10.7717/peerj.19978 (PMC12422262; doi:10.7717/peerj.19978)
Supplement: Supplemental Information 1 [file peerj-13-19978-s001.zip › Supplemental Raw Data/Supplemental-information.docx]

Table S1 qRT-PCR primers for expression analysis of *SHMT* gene family in four Salsoleae species

| Gene name | Primer sequence (5^，^to 3^，^) |  |
| --- | --- | --- |
| *SjSHMT1* | F:TATGGCTGTTGTGTTTTCCTCG | R:TGCATAGGCACTTGCTCCAG |
| *SjSHMT2* | F:CAGGAAGGGTGTTAAGGAGATCAA | R:ATGCAACAGCTAAGCCAGTGA |
| *SjSHMT4* | F:AAATCTCGTCCGCATTTCATCA | R:GAAGTGTTGCCCCATGAGTT |
| *SjSHMT7* | F:GCAACTGCGAATCCACCAAA | R:CAATTTTGCGCCGTTTGCTC |
| *OlSHMT1* | F:AAAGCCTATTCGCAACGCCT | R:TCCTCGAGCGGAGCATTCAA |
| *OlSHMT2* | F:CTGAGAATTTTACATCTGCATCGG | R:GCCCGCTTCTGACACAATGA |
| *OlSHMT3* | F:TGCCGAAAACAAAGCTGCAT | R:CAACGGGAACTGAGTGGTCA |
| *OlSHMT4* | F:CCTCCGCATTTCATCAAACACC | R:GGGAAGTGTTGCCCCATGAA |
| *OlSHMT7* | F:GATACGGATGGTGCTGACGA | R:CAGGCACATCGAATGCCCTA |
| *SfSHMT1* | F:CCTCAATGCCGCTCCAGTC | R:AATTGCTTCGGCCACGTCA |
| *SfSHMT2* | F:TGGGCATCTTTCACACGGTTAT | R:TTTGGCCTAAACAGAACAGCA |
| *SfSHMT4* | F:GCAAGTTCATCAGATCTCGTCC | R:TGTTCGATGAAATGCGGAGG |
| *SfSHMT7* | F:TACAGAGGGTGGTGACGAGA | R:TATCACGGCGTCGTTTCAGG |
| *XaSHMT1* | F:GCAGCCTTGAACGAGAAACA | R:TTCAAGGCCCTTCCATTGTCT |
| *XaSHMT2* | F:GCAAGCTGTTGGATCTGTC | R:CCAACTGAAATACTTCCAGTGCC |
| *XaSHMT3* | F:ATGTCGGTCACCGGTCTCTT | R:GCGACGCACATGAAAGACG |
| *XaSHMT4* | F:TGATAAGTGTGGGGCCCTCT | R:TGGACCCCTCAGACTCTTGT |
| *XaSHMT7* | F:GCGAGAAGCAGGGAATGGAG | R:CGTCGTTTCAGGCACATTGG |
| *β-actin* | F:TCCACGAAACAACCTACAACTC | R:CAGCAATACCGGGGAACAT |

Table S2 RNA-seq data information statistics and sequence identifiers

| Family | Abbr | Species | SRA | Photosynthetic type | Database |
| --- | --- | --- | --- | --- | --- |
| [Amaranthaceae](https://www.iplant.cn/info/Amaranthaceae) | *Sjun* | *Salsola junatovii* | SRR32571875 | C_3_ | Unpublished |
|  | *Sfol* | *Soda foliosa* | SRR32571874 | C_4_ | Unpublished |
|  | *Olar* | *Oreosalsola laricifolia* | SRR32571873 | C_2_ | Unpublished |
|  | *Xarb* | *Xylosalsola arbuscula* | SRR32571872 | C_4_ | Unpublished |
| Asteraceae | *Fcro* | *Flaveria cronquistii* | ERR2040669 | C_3_ | OneKP |
|  | *Fpri* | *Flaveria pringlei* | ERR2040673 | C_3_ | OneKP |
|  | *Fang* | *Flaveria angustifolia* | ERR2040662 | C_2_ | OneKP |
|  | *Fpub* | *Flaveria pubescens* | ERR2040675 | C_2_ | OneKP |
|  | *Fbid* | *Flaveria bidentis* | ERR2040665 | C_4_ | OneKP |
|  | *Fkoc* | *Flaveria kochiana* | ERR2040670 | C_4_ | OneKP |
|  | *Ftri* | *Flaveria trinervia* | ERR2040677 | C_4_ | OneKP |
| Boraginaceae | *Hcal* | *Heliotropium calcicola* | ERR2040514 | C_3_ | OneKP |
|  | *Hkar* | *Heliotropium karwinskyi* | ERR2040519 | C_3_ | OneKP |
|  | *Hgre* | *Heliotropium greggii* | ERR2040518 | C_2_ | OneKP |
|  | *Hrac* | *Heliotropium racemosum* | ERR2040521 | C_2_ | OneKP |
|  | *Hten* | *Heliotropium tenuifolium* | ERR2040512 | C_4_ | OneKP |
|  | *Htex* | *Heliotropium texanum* | ERR2040524 | C_4_ | OneKP |
| Molluginaceae | *Mpen* | *Mollugo pentaphylla* | ERR2040238 | C_3_ | OneKP |
|  | *Mnud* | *Mollugo nudicaulis* | ERR2040237 | C_2_ | OneKP |
|  | *Mver* | *Mollugo verticillata* | ERR2040240 | C_2_ | OneKP |
|  | *Mcer* | *Mollugo cerviana* | ERR2040235 | C_4_ | OneKP |
| Amaranthaceae | *Ases* | *Alternanthera sessilis* | ERR2040219 | C_3_ | OneKP |
|  | *Abra* | *Alternanthera brasiliana* | ERR2040215 | C_3_ | OneKP |
|  | *Aten* | *Alternanthera tenella* | ERR2040222 | C_2_ | OneKP |
|  | *Acar* | *Alternanthera caracasana* | ERR2040217 | C_4_ | OneKP |
| Poaceae | *Nalo* | *Neurachne alopecuroideae* | ERR3823069 | C_3_ | NCBI |
|  | *Nann* | *Neurachne annularis* | ERR3823071 | C_3_ | NCBI |
|  | *Nlan* | *Neurachne lanigera* | ERR3823074 | C_3_ | NCBI |
|  | *Nmin* | *Neurachne minor* | ERR3823079 | C_2_ | NCBI |
|  | *Nmue* | *Neurachne muelleri* | ERR3823081 | C_4_ | NCBI |
|  | *Nmun* | *Neurachne munroi* | ERR3823084 | C_4_ | NCBI |
| *OneKP(sshttps://db.cngb.org/onekp/);NCBI(https://www.ncbi.nlm.nih.gov/) | | | | | |

Table S3 Conserved protein domains of 18 *SHMT* gene family proteins in four Salsoleae species

| Query | Hit type | From | To | Bitscore | Accession | Superfamily |
| --- | --- | --- | --- | --- | --- | --- |
| *ts3g08640_T02* | specific | 42 | 539 | 986.015 | PLN03226 | cl18945 |
| *ts3g08640_T02* | specific | 54 | 475 | 761.596 | pfam00464 | cl18945 |
| *ts2g04227_T01* | specific | 39 | 514 | 995.26 | PLN03226 | cl18945 |
| *ts2g04227_T01* | specific | 52 | 449 | 774.307 | pfam00464 | cl18945 |
| *ts2g06369_T02* | specific | 67 | 540 | 1029.93 | PLN03226 | cl18945 |
| *ts2g06369_T02* | specific | 81 | 481 | 681.089 | pfam00464 | cl18945 |
| *ts3g08322_T02* | non-specific | 145 | 613 | 839.254 | PLN03226 | cl18945 |
| *ts3g08322_T02* | non-specific | 157 | 562 | 606.36 | pfam00464 | cl18945 |
| *sy2g05957_T04* | specific | 42 | 515 | 991.408 | PLN03226 | cl18945 |
| *sy2g05957_T04* | specific | 54 | 451 | 768.144 | pfam00464 | cl18945 |
| *sy1g02520_T01* | specific | 40 | 515 | 991.793 | PLN03226 | cl18945 |
| *sy1g02520_T01* | specific | 53 | 450 | 772.381 | pfam00464 | cl18945 |
| *sy8g29094_T01* | non-specific | 105 | 458 | 635.098 | PLN03226 | cl18945 |
| *sy8g29094_T01* | non-specific | 105 | 402 | 493.497 | pfam00464 | cl18945 |
| *sy1g00078_T01* | specific | 67 | 540 | 1029.54 | PLN03226 | cl18945 |
| *sy1g00078_T01* | specific | 81 | 481 | 680.704 | pfam00464 | cl18945 |
| *sy2g06541_T03* | non-specific | 145 | 613 | 839.639 | PLN03226 | cl18945 |
| *sy2g06541_T03* | non-specific | 157 | 562 | 604.049 | pfam00464 | cl18945 |
| *jg1g00713_T01* | specific | 43 | 515 | 988.711 | PLN03226 | cl18945 |
| *jg1g00713_T01* | specific | *54* | *451* | *767.759* | pfam00464 | cl18945 |
| *jg2g04576_T01* | specific | 41 | 516 | 994.489 | PLN03226 | cl18945 |
| *jg2g04576_T01* | specific | 54 | 451 | 773.152 | pfam00464 | cl18945 |
| *jg2g02805_T02* | specific | 67 | 540 | 1032.24 | PLN03226 | cl18945 |
| *jg2g02805_T02* | specific | 81 | 481 | 680.704 | pfam00464 | cl18945 |
| *jg1g01220_T02* | non-specific | 145 | 613 | 841.95 | PLN03226 | cl18945 |
| *jg1g01220_T02* | non-specific | 157 | 562 | 605.205 | pfam00464 | cl18945 |
| *mbsg23959_T02* | specific | 39 | 512 | 1002.19 | PLN03226 | cl18945 |
| *mbsg23959_T02* | specific | 51 | 448 | 774.692 | pfam00464 | cl18945 |
| *mb1g00716_T01* | specific | 42 | 517 | 995.26 | PLN03226 | cl18945 |
| *mb1g00716_T01* | specific | 55 | 452 | 769.3 | pfam00464 | cl18945 |
| *mb5g11984_T01* | specific | 76 | 532 | 905.508 | PLN03226 | cl18945 |
| *mb5g11984_T01* | specific | 86 | 476 | 711.135 | pfam00464 | cl18945 |
| *mb4g10902_T02* | specific | 68 | 541 | 1030.31 | PLN03226 | cl18945 |
| *mb4g10902_T02* | specific | 82 | 482 | 681.859 | pfam00464 | cl18945 |
| *mb5g13083_T01* | non-specific | 143 | 613 | 836.558 | PLN03226 | cl18945 |
| *mb5g13083_T01* | non-specific | 160 | 562 | 600.967 | pfam00464 | cl18945 |

Table S4 Confidence scores for the AlphaFold3-predicted three-dimensional structures of SHMT proteins.

| Species | ipTM | pTM |
| --- | --- | --- |
| *SjSHMT1* | 0.84 | 0.86 |
| *SjSHMT2* | 0.87 | 0.88 |
| *SjSHMT4* | 0.84 | 0.85 |
| *SjSHMT7* | 0.76 | 0.77 |
| *OlSHMT1* | 0.87 | 0.88 |
| *OlSHMT2* | 0.88 | 0.89 |
| *OlSHMT3* | 0.29 | 0.36 |
| *OlSHMT4* | 0.84 | 0.85 |
| *OlSHMT7* | 0.76 | 0.77 |
| *SfSHMT1* | 0.87 | 0.89 |
| *SfSHMT2* | 0.87 | 0.88 |
| *SfSHMT4* | 0.84 | 0.85 |
| *SfSHMT7* | 0.75 | 0.76 |
| *XaSHMT1* | 0.87 | 0.88 |
| *XaSHMT2* | 0.86 | 0.88 |
| *XaSHMT3* | 0.82 | 0.83 |
| *XaSHMT4* | 0.84 | 0.85 |

Note: Although two confidence metrics for OlSHMT3 are relatively low, likely because, despite its intact SHMT domain, some other sequence regions are missing, we nonetheless included it as a member in our analysis.

Table S5 Details of the 20 conserved motifs in SHMT proteins of Salsoleae species

| Motif | Width | Motif Sequence |
| --- | --- | --- |
| Motif1 | 50 | TNKYSEGYPGARYYGGNEYIDMIETLCQKRALEAFRLDPEKWGVNVQPLS |
| Motif2 | 50 | LADMAHISGLVAAGVIPSPFEYCDIVTTTTHKSLRGPRGGMIFFRKGVKP |
| Motif3 | 50 | YDFEDKINFAVFPGLQGGPHNHTIAALAVALKQAMTPEYKAYQQQVLSNA |
| Motif4 | 50 | YKLBESTGYIDYDKLEKKALLFRPKLIIAGASAYPRLYDYARIRKIADKC |
| Motif5 | 43 | SLEEIDPEIADIIEKEKARQWKGJELIASENFTSKAVMZAVGS |
| Motif6 | 41 | GSPANFAVYTALLKPHERIMGLDLPHGGHLSHGYYTDTKKI |
| Motif7 | 50 | GTDNHLVLVBLRPLGJDGSRVEKVCEAVHIALNKNAVPGDVSAMVPGGIR |
| Motif8 | 41 | MGTPAMTSRGFIEEDFEKVAEFLDAAVKLALKIKAETGKTK |
| Motif9 | 41 | AAFNKSGVFNSEIEKLRAEVEEYAKQFPTIGFEKESMKYKD |
| Motif10 | 11 | SAVSIFFETMP |
| Motif11 | 50 | TANPPNPNRISINDDSISFQIDSRLRESSNPISSIPLQLLEQTAQNCRKM |
| Motif12 | 21 | SQPIRPPFNSAPHYYMSSVAN |
| Motif13 | 50 | FRILGHPMCLKRRRDREDSVSSSSADSAKRMFFDSSLELRRCAVRSWGNQ |
| Motif14 | 29 | CFLKGLPKNDDIVELRPRIEVMEAQFSMP |
| Motif15 | 15 | FAEALLSKGYELVSG |
| Motif16 | 15 | IEKRSSVTWPKQLNE |
| Motif17 | 11 | MAMTMALRKLS |
| Motif18 | 29 | DKDRVSENTKDTDGADERGAVNGDEEVEE |
| Motif19 | 21 | MQVQQISSSSTAVYNFKGRKV |
| Motif20 | 8 | INKKGKEV |

Table S6 *SHMT* members of all species used for phylogenetic tree construction.

| Species | Gene names | Gene ID | Data sources |
| --- | --- | --- | --- |
| *Arabidopsis*  *thaliana* | *AtSHMT1* | *AT4G37930* | Phytozome  TAIR |
|  | *AtSHMT2* | *AT5G26780* |  |
|  | *AtSHMT3* | *AT4G32520* |  |
|  | *AtSHMT4* | *AT4G13930* |  |
|  | *AtSHMT5* | *AT4G13890* |  |
|  | *AtSHMT6* | *AT1G22020* |  |
|  | *AtSHMT7* | *AT1G36370* |  |
| *Glycine max* | *GmSHMT02m* | *Glyma.02G217100* | (*Lakhssassi et al., 2019*) |
|  | *GmSHMT04n* | *Glyma.04G254300* |  |
|  | *GmSHMT05c* | *Glyma.05G152100* |  |
|  | *GmSHMT06n* | *Glyma.06G107800* |  |
|  | *GmSHMT08c* | *Glyma.08G108900* |  |
|  | *GmSHMT08m* | *Glyma.08G274400* |  |
|  | *GmSHMT08n* | *Glyma.08G187800* |  |
|  | *GmSHMT09m* | *Glyma.09G202000* |  |
|  | *GmSHMT09n* | *Glyma.09G184300* |  |
|  | *GmSHMT12c* | *Glyma.12G159200* |  |
|  | *GmSHMT12n* | *Glyma.12G170300* |  |
|  | *GmSHMT13c* | *Glyma.13G077700* |  |
|  | *GmSHMT13ch* | *Glyma.13G222300* |  |
|  | *GmSHMT14m* | *Glyma.14G184500* |  |
|  | *GmSHMT15c* | *Glyma.15G090000* |  |
|  | *GmSHMT15ch* | *Glyma.15G089900* |  |
|  | *GmSHMT16c* | *Glyma.16G108100* |  |
|  | *GmSHMT18m* | *Glyma.18G150000* |  |
| *Solanum lycopersicum* | *SlSHMT1* | *Solyc01g104000.3.1* | Phytozome |
|  | *SlSHMT2* | *Solyc02g091560.3.1* |  |
|  | *SlSHMT3* | *Solyc04g076790.3.1* |  |
|  | *SlSHMT4* | *Solyc05g053810.3.1* |  |
|  | *SlSHMT5* | *Solyc08g065490.3.1* |  |
|  | *SlSHMT6* | *Solyc12g095930.2.1* |  |
|  | *SlSHMT7* | *Solyc12g098490.2.1* |  |
| *Populus trichocarpa* | *PtrSHMT1* | *Potri.001G212000.3* | Phytozome  (*Bing et al.,2020*) |
|  | *PtrSHMT2* | *Potri.001G320400.2* |  |
|  | *PtrSHMT3* | *Potri.002G090200.1* |  |
|  | *PtrSHMT4* | *Potri.002G109200.5* |  |
|  | *PtrSHMT5* | *Potri.005G170800.1* |  |
|  | *PtrSHMT6* | *Potri.006G232300.2* |  |
|  | *PtrSHMT7* | *Potri.008G002900.14* |  |
|  | *PtrSHMT8* | *Potri.010G254700.15* |  |
|  | *PtrSHMT9* | *Potri.017G059300.1* |  |
| *Cucumis sativus* | *CsSHMT1* | *CsaV3_6G044790.1* | CuGenDB |
|  | *CsSHMT2* | *CsaV3_2G030460.1* |  |
|  | *CsSHMT3* | *CsaV3_4G007440.1* |  |
|  | *CsSHMT4* | *CsaV3_2G011710.1* |  |
|  | *CsSHMT5* | *CsaV3_4G028640.1* |  |
|  | *CsSHMT6* | *CsaV3_3G019180.1* |  |
| *Beta vulgaris* | *BvSHMT1* | *EL10Ac6g13310.1* | Phytozome |
|  | *BvSHMT2* | *EL10Ac1g00464.1* |  |
|  | *BvSHMT3* | *EL10Ac2g03923.1* |  |
|  | *BvSHMT4* | *EL10Ac3g07453.1* |  |
|  | *BvSHMT7* | *EL10Ac6g14068.1* |  |
| *Oryza sativa* | *OsSHMT1* | *Os03t0738400-01* | Phytozome |
|  | *OsSHMT2* | *Os11t0455800-01* |  |
|  | *OsSHMT3* | *Os12t0409000-01* |  |
|  | *OsSHMT4* | *Os01t0874900-01* |  |
|  | *OsSHMT5* | *Os05t0429000-01* |  |
| *Salsola junatovii* | *SjSHMT1* | *ts3g08640_T02* | - |
|  | *SjSHMT2* | *ts2g04227_T01* |  |
|  | *SjSHMT3* | *ts9g25194_T01* |  |
|  | *SjSHMT4* | *ts2g06369_T02* |  |
|  | *SjSHMT7* | *ts3g08322_T02* |  |
| *Oreosalsola laricifolia* | *OlSHMT1* | *sy2g05957_T04* | - |
|  | *OlSHMT2* | *sy1g02520_T01* |  |
|  | *OlSHMT3* | *sy8g29094_T01* |  |
|  | *OlSHMT4* | *sy1g00078_T01* |  |
|  | *OlSHMT7* | *sy2g06541_T03* |  |
| *Soda foliosa* | *SfSHMT1* | *jg1g00713_T01* | - |
|  | *SfSHMT2* | *jg2g04576_T01* |  |
|  | *SfSHMT4* | *jg2g02805_T02* |  |
|  | *SfSHMT7* | *jg1g01220_T02* |  |
|  |  |  |  |
| *Xylosalsola arbuscula* | *XaSHMT1* | *mbsg23959_T02* | - |
|  | *XaSHMT2* | *mb1g00716_T01* |  |
|  | *XaSHMT3* | *mb5g11984_T01* |  |
|  | *XaSHMT4* | *mb4g10902_T02* |  |
|  | *XaSHMT7* | *mb5g13083_T01* |  |

Table S7. Collinearity relationships of *SHMT* numbers between four Salsoleae species, *Arabidopsis thaliana*, and *Beta vulgaris*

| Species | Gene ID | Collinear relationships |
| --- | --- | --- |
| *Salsola junatovii* | *Sj-ts3g08640_T02(SjSHMT1)* | *At-AT4G37930.1.Araport11.447(AtSHMT1)* |
|  | *Sj-ts3g08640_T02(SjSHMT1)* | *Ol-sy2g05957_T04(OlSHMT1)* |
|  | *Sj-ts3g08640_T02(SjSHMT1)* | *Sf-jg1g00713_T03(SfSHMT1)* |
|  | *Sj-ts3g08640_T02(SjSHMT1)* | *Xa-mbsg23959_T04(XaSHMT1)* |
|  | *Sj-ts3g08640_T02(SjSHMT1)* | *Bv-EL10Ac6g13310.1.EL10_1.0(BvSHMT1)* |
|  | *Sj-ts2g04227_T01(SjSHMT2)* | *Ol-sy1g02520_T01(OlSHMT2)* |
|  | *Sj-ts2g04227_T01(SjSHMT2)* | *Sf-jg2g04576_T01(SfSHMT2)* |
|  | *Sj-ts2g04227_T01(SjSHMT2)* | *Xa-mb1g00716_T01(XaSHMT2)* |
|  | *Sj-ts2g04227_T01(SjSHMT2)* | *Bv-EL10Ac1g00464.1.EL10_1.0(BvSHMT2)* |
|  | *Sj-ts2g06369_T02(SjSHMT4)* | *At-AT4G13890.1.Araport11.447(AtSHMT5)* |
|  | *Sj-ts2g06369_T02(SjSHMT4)* | *Ol-sy1g00078_T01(OlSHMT4)* |
|  | *Sj-ts2g06369_T02(SjSHMT4)* | *Xa-mb4g10902_T02(XaSHMT4)* |
|  | *Sj-ts2g06369_T02(SjSHMT4)* | *Bv-EL10Ac3g07453.1.EL10_1.0(BvSHMT4)* |
|  | *Sj-ts3g08322_T02(SjSHMT7)* | *Bv-EL10Ac6g14068.1.EL10_1.0(BvSHMT7)* |
| *Oreosalsola laricifolia* | *Ol-sy2g05957_T04(OlSHMT1)* | *At-AT4G37930.1.Araport11.447(AtSHMT1)* |
|  | *Ol-sy2g05957_T04(OlSHMT1)* | *Sj-ts3g08640_T02(SjSHMT1)* |
|  | *Ol-sy2g05957_T04(OlSHMT1)* | *Sf-jg1g00713_T03(SfSHMT1)* |
|  | *Ol-sy2g05957_T04(OlSHMT1)* | *Xa-mbsg23959_T04(XaSHMT1)* |
|  | *Ol-sy2g05957_T04(OlSHMT1)* | *Bv-EL10Ac6g13310.1.EL10_1.0(BvSHMT1)* |
|  | *Ol-sy1g02520_T01(OlSHMT2)* | *Sj-ts2g04227_T01(SjSHMT2)* |
|  | *Ol-sy1g02520_T01(OlSHMT2)* | *Sf-jg2g04576_T01(SfSHMT2)* |
|  | *Ol-sy1g02520_T01(OlSHMT2)* | *Xa-mb1g00716_T01(XaSHMT2)* |
|  | *Ol-sy1g02520_T01(OlSHMT2)* | *Bv-EL10Ac1g00464.1.EL10_1.0(BvSHMT2)* |
|  | *Ol-sy8g29094_T01(OlSHMT3)* | *Xa-mb5g11984_T02(XaSHMT3)* |
|  | *Ol-sy8g29094_T01(OlSHMT3)* | *Bv-EL10Ac2g03923.1.EL10_1.0(BvSHMT3)* |
|  | *Ol-sy1g00078_T01(OlSHMT4)* | *At-AT4G13890.1.Araport11.447(AtSHMT5)* |
|  | *Ol-sy1g00078_T01(OlSHMT4)* | *Sj-ts2g06369_T02(SjSHMT4)* |
|  | *Ol-sy1g00078_T01(OlSHMT4)* | *Sf-jg2g02805_T02(SfSHMT4)* |
|  | *Ol-sy1g00078_T01(OlSHMT4)* | *Xa-mb4g10902_T02(XaSHMT4)* |
|  | *Ol-sy1g00078_T01(OlSHMT4)* | *Bv-EL10Ac3g07453.1.EL10_1.0(BvSHMT4)* |
| *Soda foliosa* | *Sf-jg2g04576_T01(SfSHMT2)* | *Sj-ts2g04227_T01(SjSHMT2)* |
|  | *Sf-jg2g04576_T01(SfSHMT2)* | *Ol-sy1g02520_T01(OlSHMT2)* |
|  | *Sf-jg2g04576_T01(SfSHMT2)* | *Xa-mb1g00716_T01(XaSHMT2)* |
|  | *Sf-jg2g04576_T01(SfSHMT2)* | *Bv-EL10Ac1g00464.1.EL10_1.0(BvSHMT2)* |
|  | *Sf-jg2g02805_T02(SfSHMT4)* | *At-AT4G13890.1.Araport11.447(AtSHMT5)* |
|  | *Sf-jg2g02805_T02(SfSHMT4)* | *Sj-ts2g06369_T02(SjSHMT4)* |
|  | *Sf-jg2g02805_T02(SfSHMT4)* | *Ol-sy1g00078_T01(OlSHMT4)* |
|  | *Sf-jg2g02805_T02(SfSHMT4)* | *Xa-mb4g10902_T02(XaSHMT4)* |
|  | *Sf-jg2g02805_T02(SfSHMT4)* | *Bv-EL10Ac3g07453.1.EL10_1.0(BvSHMT4)* |
| *Xylosalsola arbuscula* | *Xa-mb1g00716_T01(XaSHMT2)* | *Sj-ts2g04227_T01(SjSHMT2)* |
|  | *Xa-mb1g00716_T01(XaSHMT2)* | *Ol-sy1g02520_T01(OlSHMT2)* |
|  | *Xa-mb1g00716_T01(XaSHMT2)* | *Sf-jg2g04576_T01(SfSHMT2)* |
|  | *Xa-mb1g00716_T01(XaSHMT2)* | *Bv-EL10Ac1g00464.1.EL10_1.0(BvSHMT2)* |
|  | *Xa-mb4g10902_T02(XaSHMT4)* | *At-AT4G13890.1.Araport11.447(AtSHMT5)* |
|  | *Xa-mb4g10902_T02(XaSHMT4)* | *Sj-ts2g06369_T02(SjSHMT4)* |
|  | *Xa-mb4g10902_T02(XaSHMT4)* | *Ol-sy1g00078_T01(OlSHMT4)* |
|  | *Xa-mb4g10902_T02(XaSHMT4)* | *Sf-jg2g02805_T02(SfSHMT4)* |
|  | *Xa-mb4g10902_T02(XaSHMT4)* | *Bv-EL10Ac3g07453.1.EL10_1.0(BvSHMT4)* |


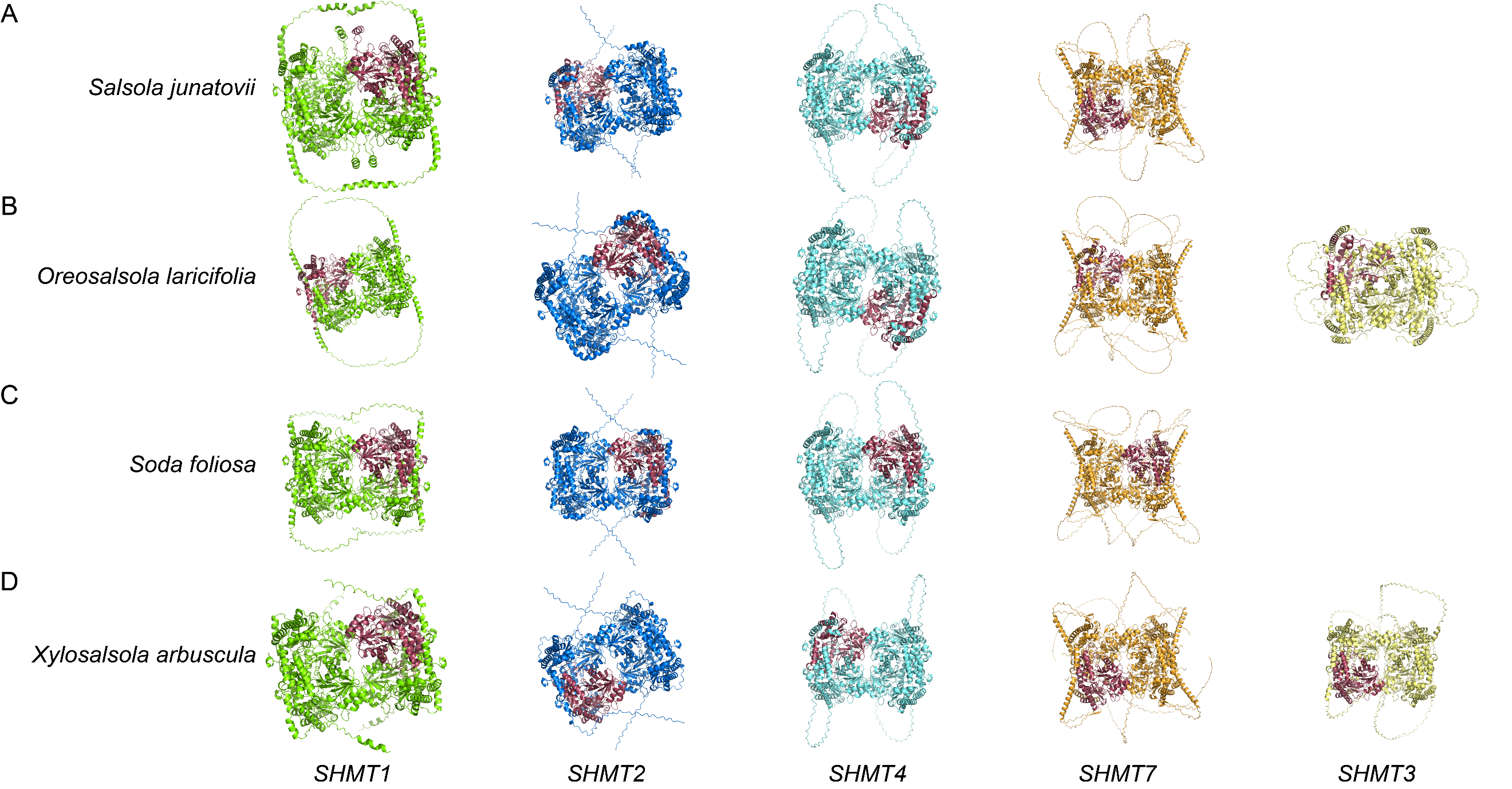


Fig. S1 Three-dimensional structures of SHMT proteins from four Salsoleae species, with the rose-red coloring indicating the conserved SHMT domain (Pfam00464).

| 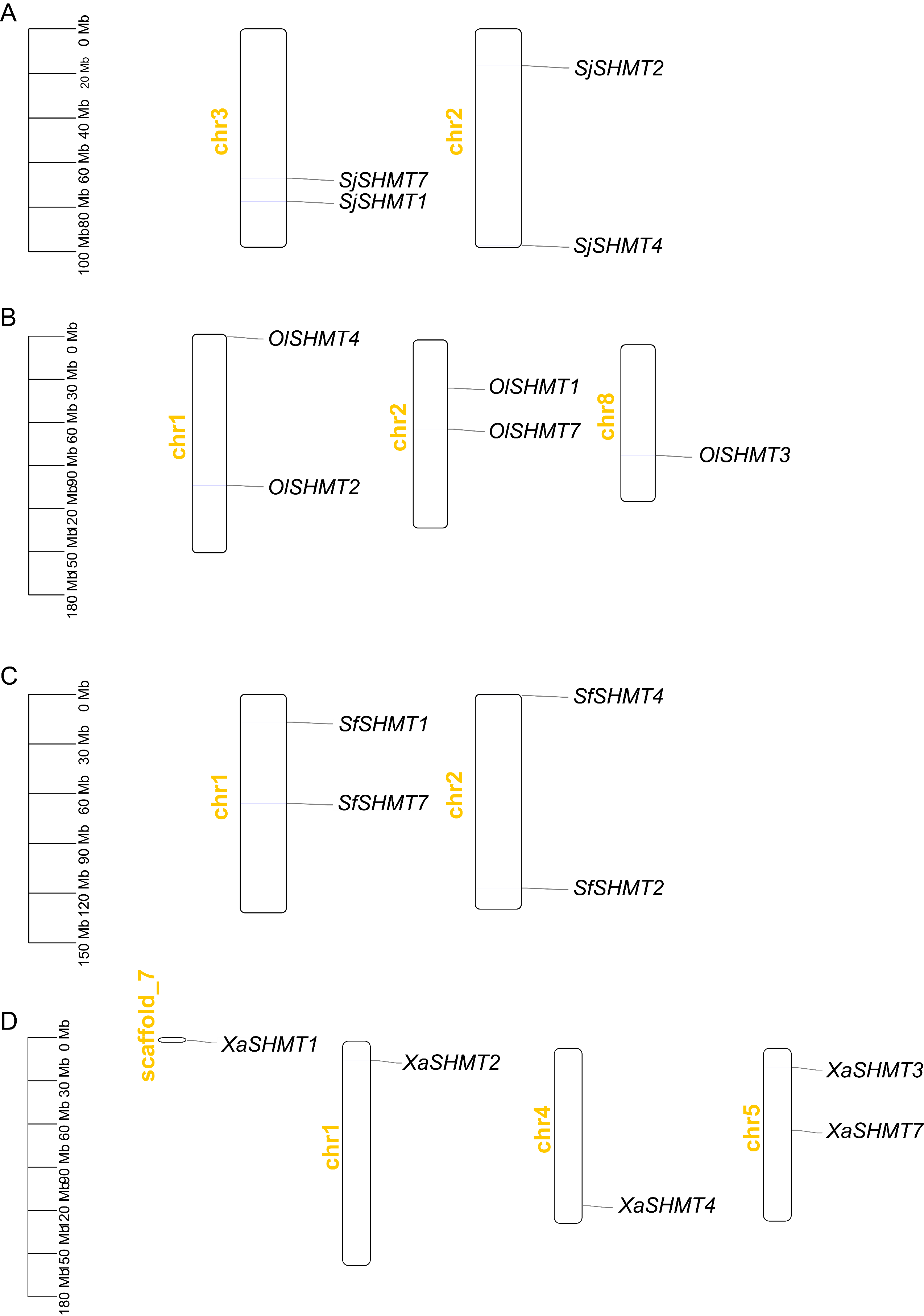 |
| --- |
| Fig. S2 Chromosomal positioning and distribution of *SHMTs* in four Salsoleae species. |

| A | 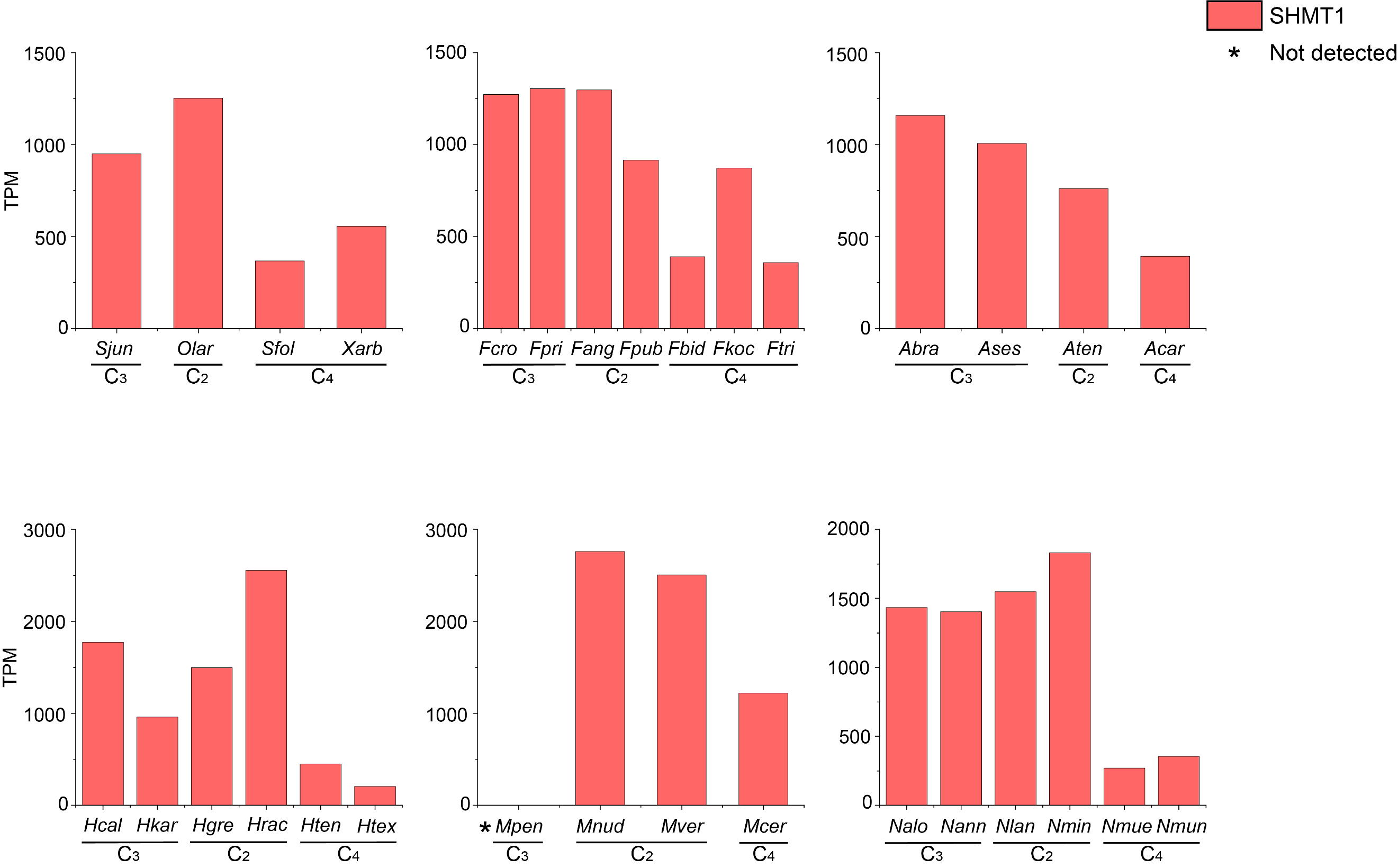 |
| --- | --- |
| B | 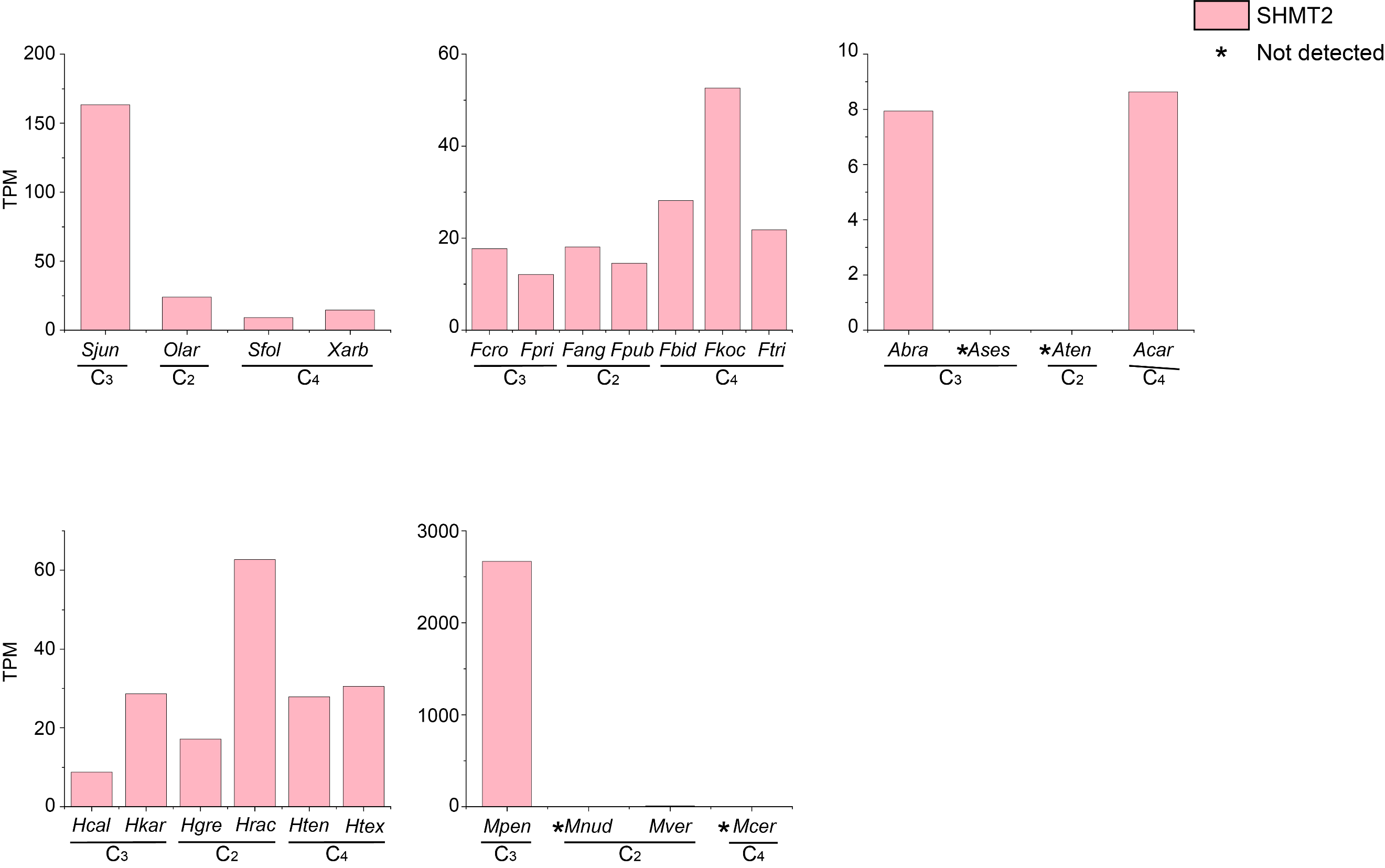 |
| C | 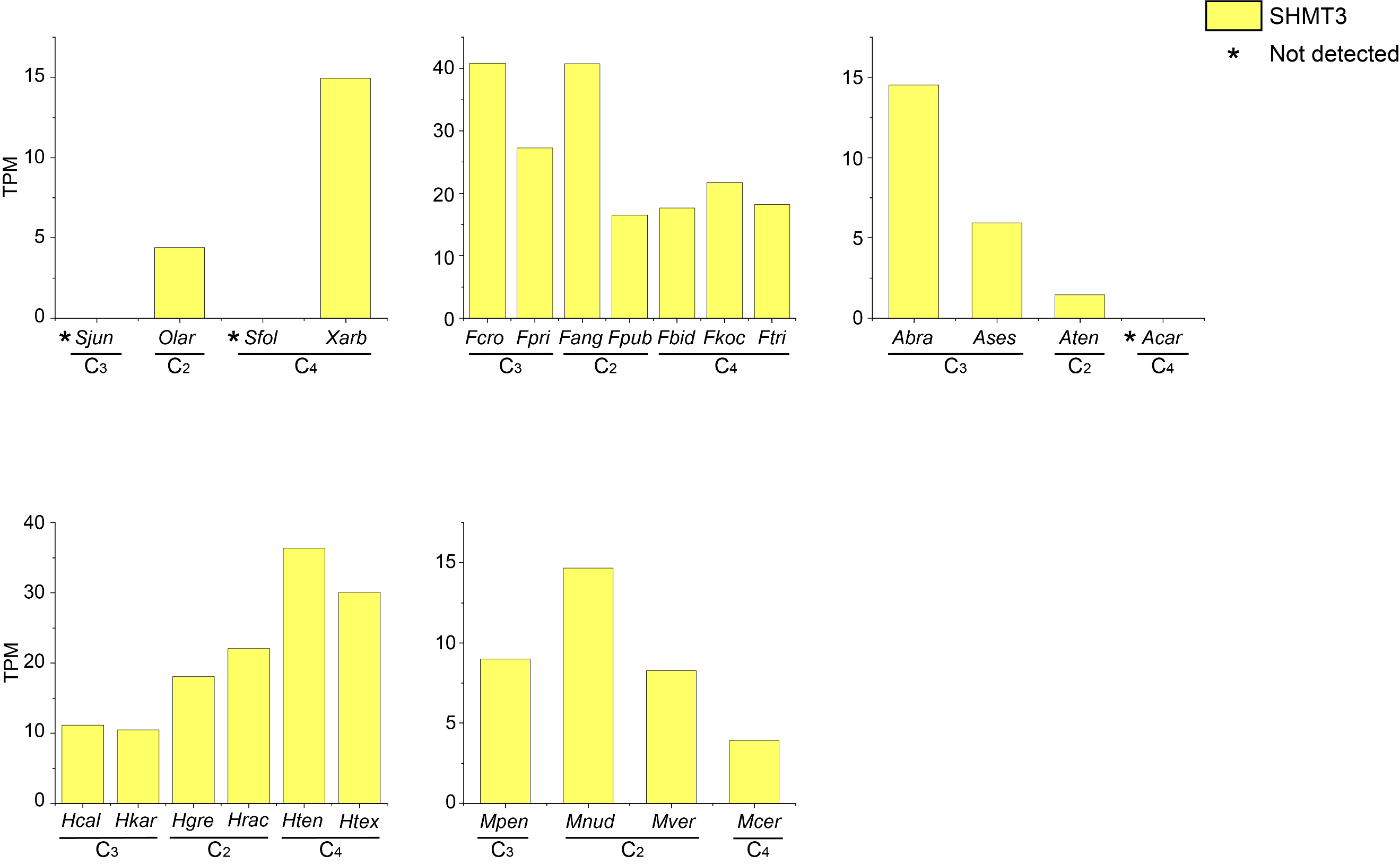 |
| D | 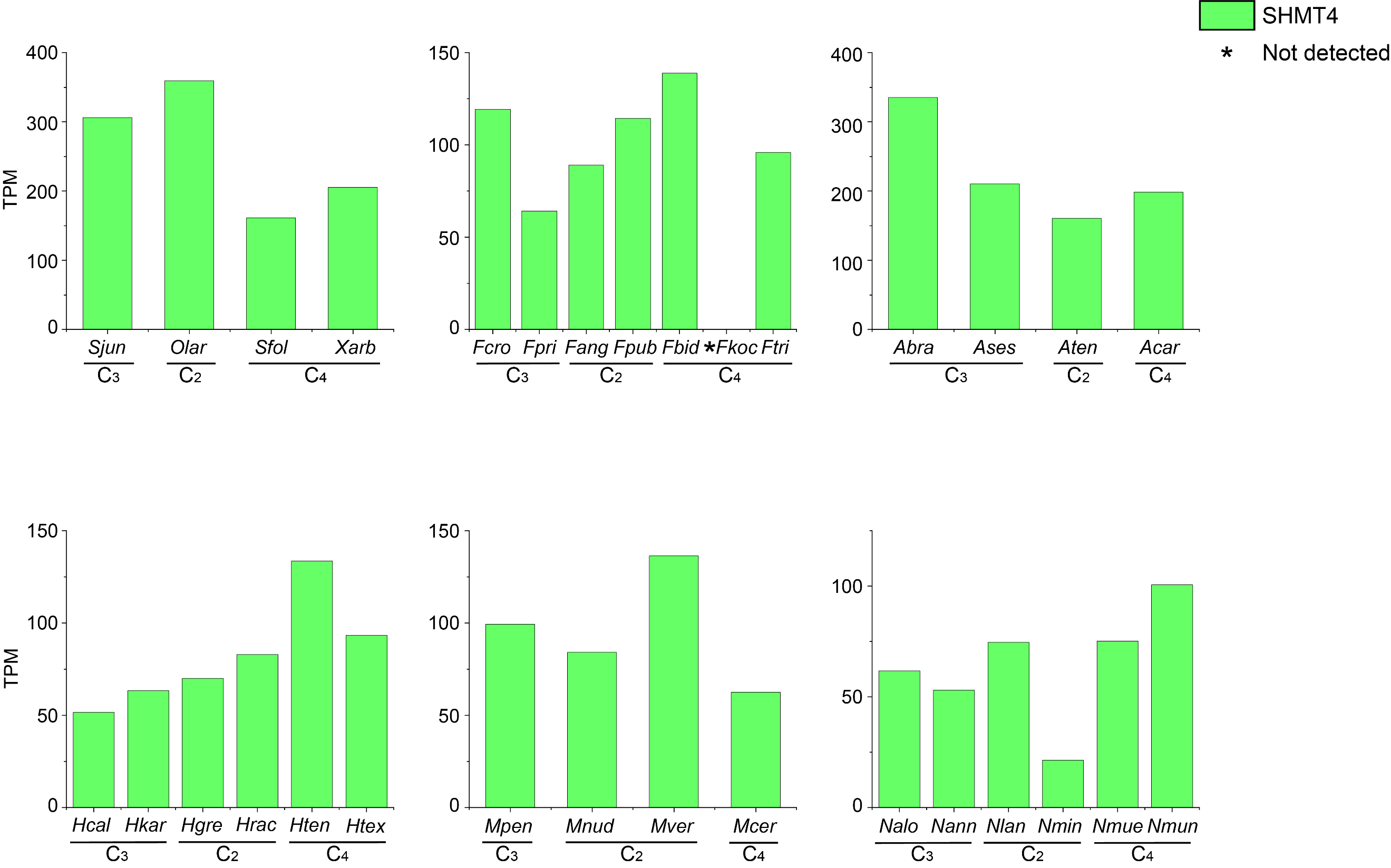 |
| E | 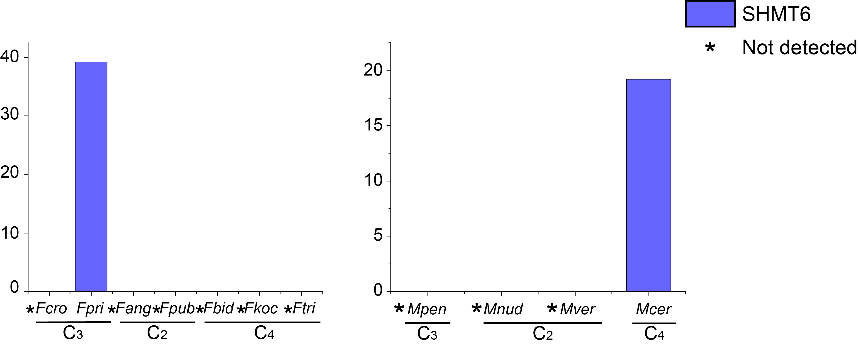 |
| F | 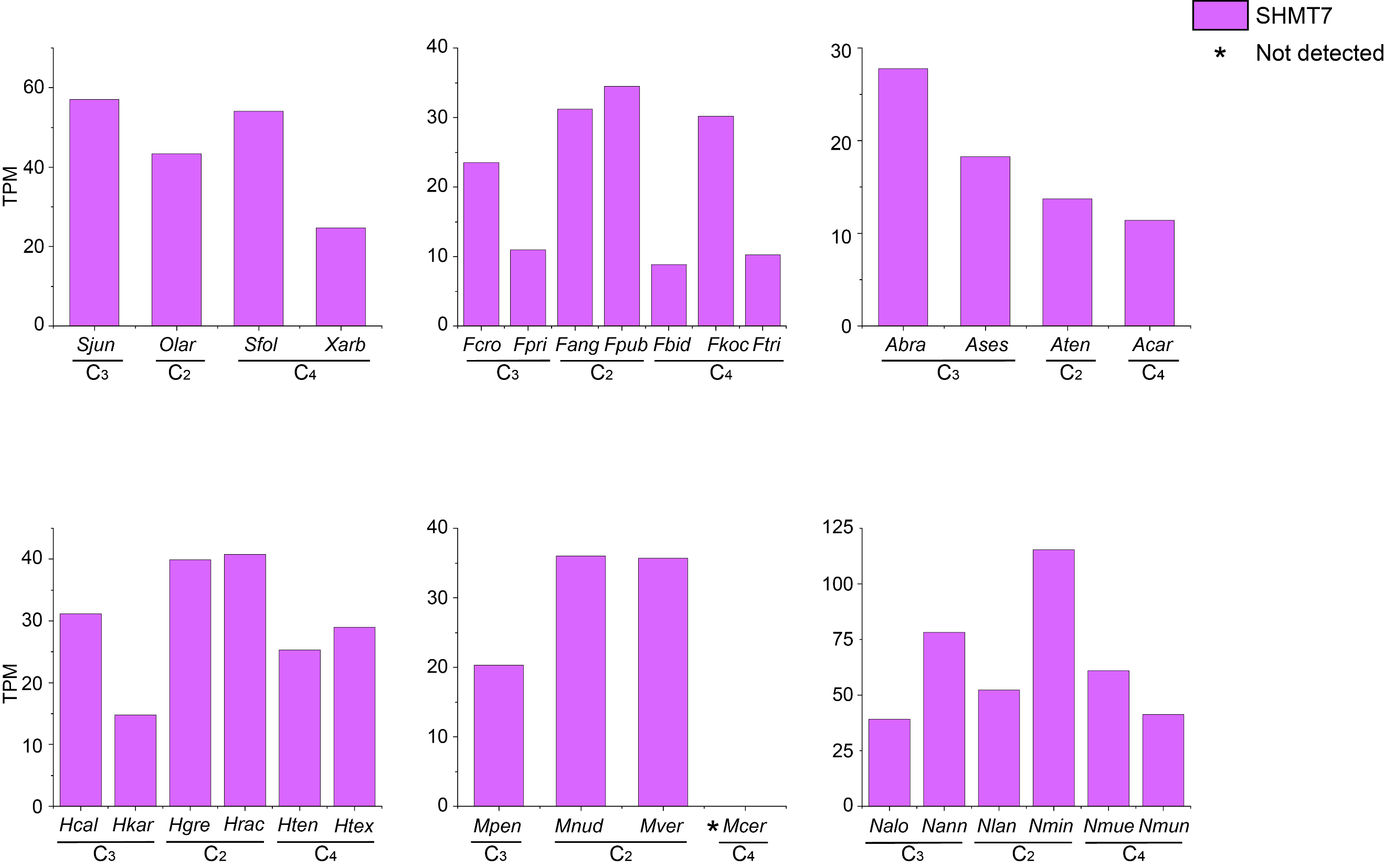 |
| Fig. S3. Expression levels of SHMTs in leaves from six different plant groups (A–F). The abbreviations of the species are as follows: *Sjun: Salsola junatovii*, *Ola: Oreosalsola laricifolia*, s*fol: Soda foliosa*, *Xarb: Xylosalsola arbuscula*; *Mpen*: *Mollugo pentaphylla*, *Mnud*: *Mollugo* *nudicaulis*, *Mver*: *Mollugo* *verticillate*, *Mcer*: *Mollugo* *cerviana*, *Ases*: *Alternanthera* *sessilis*, *Abra*: *Alternanthera* *brasiliana*, *Aten*: *Alternanthera* *tenella*, *Acar*: *Alternanthera* *caracasana*, *Fcro*: *Flaveria* *cronquistii*, *Fpri*: *Flaveria* *pringlei*, *Fang*: *Flaveria* *angustifolia*, *Fpub*: *Flaveria* *pubescens*, *Fbid*: *Flaveria* *bidentis*, *Fkoc*: *Flaveria* *kochiana*, *Ftri*: *Flaveria* *trinitrin*, *Hcal*: *Heliotropium* *calcicola*, *Hkar*: *Heliotropium* *karwinskyi*, *Hgre*: *Heliotropium* *greggii*, *Hrac*: *Heliotropium* *racemosum*, *Hten*: *Heliotropium* *tenuifolium*, *Htex*: *Heliotropium* *texanum*; *Nalo*: *Neurachne* *alopecuroideae*, *Nann*: *Neurachne* *annularis*, *Nlan*: *Neurachne* *lanigera*, *Nmin*: *Neurachne* *minor*, *Nmun*: *Neurachne* *munroi*, *Nmue*: *Neurachne* *muelleri*, *Nmun*: *Neurachne* *munroi.* | |
